# The spatio-temporal distribution of acute encephalitis syndrome and its association with climate and landcover in Vietnam

**DOI:** 10.1186/s12879-023-08300-1

**Published:** 2023-06-13

**Authors:** Hannah E. Brindle, Leonardo S. Bastos, Robert Christley, Lucie Contamin, Le Hai Dang, Dang Duc Anh, Neil French, Michael Griffiths, Behzad Nadjm, H. Rogier van Doorn, Pham Quang Thai, Tran Nhu Duong, Marc Choisy

**Affiliations:** 1 Institute of Infection, Veterinary and Ecological Sciences, University of Liverpool, Liverpool, UK; 2 Oxford University Clinical Research Unit, Hanoi City, Vietnam; 3 Scientific Computing Programme, Oswaldo Cruz Foundation, Rio de Janeiro, Brazil; 4 Institut de Recherche Pour Le Développement, Hanoi, Vietnam; 5 National Institute of Hygiene and Epidemiology, Hanoi, Vietnam; 6 MRC Unit The Gambia at the London, School of Hygiene & Tropical Medicine, Fajara, The Gambia; 7 Centre for Tropical Medicine and Global Health, Nuffield Department of Medicine, University of Oxford, Oxford, UK; 8 School Preventive Medicine and Public Health, Hanoi Medical University, Hanoi, Vietnam; 9 Oxford University Clinical Research Unit, Ho Chi Minh City, Vietnam

**Keywords:** Vietnam, Encephalitis, Spatio-temporal, Climate, Vegetation, Vaccination

## Abstract

**Background:**

Acute encephalitis syndrome (AES) differs in its spatio-temporal distribution in Vietnam with the highest incidence seen during the summer months in the northern provinces. AES has multiple aetiologies, and the cause remains unknown in many cases. While vector-borne disease such as Japanese encephalitis and dengue virus and non-vector-borne diseases such as influenza and enterovirus show evidence of seasonality, associations with climate variables and the spatio-temporal distribution in Vietnam differs between these. The aim of this study was therefore to understand the spatio-temporal distribution of, and risk factors for AES in Vietnam to help hypothesise the aetiology.

**Methods:**

The number of monthly cases per province for AES, meningitis and diseases including dengue fever; influenza-like-illness (ILI); hand, foot, and mouth disease (HFMD); and *Streptococcus suis* were obtained from the General Department for Preventive Medicine (GDPM) from 1998–2016. Covariates including climate, normalized difference vegetation index (NDVI), elevation, the number of pigs, socio-demographics, JEV vaccination coverage and the number of hospitals were also collected. Spatio-temporal multivariable mixed-effects negative binomial Bayesian models with an outcome of the number of cases of AES, a combination of the covariates and harmonic terms to determine the magnitude of seasonality were developed.

**Results:**

The national monthly incidence of AES declined by 63.3% over the study period. However, incidence increased in some provinces, particularly in the Northwest region. In northern Vietnam, the incidence peaked in the summer months in contrast to the southern provinces where incidence remained relatively constant throughout the year. The incidence of meningitis, ILI and *S. suis* infection; temperature, relative humidity with no lag, NDVI at a lag of one month, and the number of pigs per 100,000 population were positively associated with the number of cases of AES in all models in which these covariates were included.

**Conclusions:**

The positive correlation of AES with temperature and humidity suggest that a number of cases may be due to vector-borne diseases, suggesting a need to focus on vaccination campaigns. However, further surveillance and research are recommended to investigate other possible aetiologies such as *S. suis* or *Orientia tsutsugamushi*.

**Supplementary Information:**

The online version contains supplementary material available at 10.1186/s12879-023-08300-1.

## Background

Viral encephalitis (VE) defined as ‘fever greater than 38 °C and a change in mental status, seizures, abnormal movements, tremor or spastic paralysis is a mandatory notifiable disease in Vietnam and is used as the case definition for the surveillance of acute encephalitis syndrome (AES) [1]. A study from 1998–2007 conducted in Vietnam, showed a reduction in the national incidence of AES over the ten-year period from 3.0 to 1.4 cases per 100,000 population. The highest incidences were observed in the mountainous provinces of the northern regions with peaks in the number of cases in the summer months [2]. Vietnam has a varied terrain, ranging from the low-lying Mekong and Red River Delta regions, to mountainous areas in the regions of the Northwest, Northeast and Central Highlands [3]. In northern Vietnam there is a cool and humid winter with a warm and wet summer in contrast to the tropical climate of southern Vietnam which experiences a wet season from May to November and dry season from December to April [4–7]. A study conducted in two provinces of northern Vietnam from 2004 to 2013 identified a positive association between the incidence of VE and temperature, hours of sunshine, a relative humidity greater than 80% and increases in rainfall to a mean value of 130 mm during the preceding and current months [8]. In Vietnam and its neighbouring countries in the Mekong region, a number of different pathogens contribute to the aetiology of AES. However, the cause remains unknown in approximately one to two thirds of cases [9–12].

The epidemiology of vector-borne diseases is largely influenced by climate and vegetation. Japanese encephalitis virus (JEV), the most common viral cause of central nervous system (CNS) infections in children in Vietnam and neighbouring countries, and in adults in some studies [9–12], is transmitted in an enzootic cycle between *Culex* mosquitoes, most commonly *Culex tritaeniorhynchus*, and animals such as wild birds and pigs, with humans as the dead-end hosts [13–17]. Rice fields, marshes and swamps serve as breeding sites for *Cx. tritaeniorhynchus* larvae [18–20] with irrigated land shown to be positively associated with the incidence of Japanese encephalitis (JE) [21]. Dengue virus (DENV), another common cause of viral CNS infections in the region [9–12] is transmitted by the bite of infected *Aedes* mosquitoes, primarily *Aedes aegypti* but also *Aedes albopictus* [22], which tend to breed in water storage containers and used tyres [23–25]. The lifecycle of mosquitoes is sensitive to changes in temperature, humidity and rainfall. At temperatures too low or too high, the mosquitoes cannot breed and their activity and survival are reduced [26–30]. Insufficient rainfall and low levels of humidity can cause desiccation of the mosquito eggs, while heavy rainfall can flood breeding sites, washing away larvae [19, 28–33]. As a result of this, JEV and DENV are endemic in the southern provinces of Vietnam with cases occurring throughout the year and a peak in incidence of cases of dengue fever from July–September [34, 35]. In the northern provinces, cases of JE are seasonal with peaks in incidence in the summer months whereas numbers of cases of dengue fever are low in the winter months with Hanoi seeing peaks in incidence in the autumn [34].

Diseases which are not vector-borne may also show evidence of seasonality. In Vietnamese children, after JEV, the second most common cause of viral encephalitis in Vietnam is enterovirus including enterovirus 71 [11]. Enterovirus 71 is primarily transmitted via the faecal-oral and respiratory routes and is seen most commonly in children aged under five years with outbreaks of hand, foot, and mouth diseases (HFMD) particularly occurring in nurseries and primary schools [36–39]. Sixty percent of cases of HFMD occur in southern Vietnam with peaks in incidence from March to May and September to December [40]. *Streptococcus pneumoniae* and *Haemophilus influenzae* type b (Hib), the most common bacterial causes of CNS infections in children in Vietnam, are also transmitted via respiratory secretions [11, 41–43]. While there are no studies on the seasonality of these pathogens in Vietnam, research conducted on the Thailand-Myanmar border showed that transmissibility of *S. pneumoniae* was highest during the cool and dry months and, similarly, in Taiwan, most cases of Hib occurred during the winter [44, 45]. *Streptococcus suis*, the most common bacterial cause of CNS infections in adults in Vietnam is acquired via the consumption or handling of raw or undercooked pig products such as blood and intestines, tonsils, tongue and the uterus [43, 46–48]. In northern Vietnam, the incidence of *S. suis* has been shown to peak between May and July. However, in southern Vietnam there is no clear evidence of seasonality with cases occurring throughout the year [47, 49]. Influenza virus is a rarer cause of AES in Vietnam [11, 50] and cases of influenza-like-illness are positively associated with absolute humidity, with cases peaking in the late summer/early autumn in northern Vietnam while remaining relatively constant in the southern provinces [3].

However, not all pathogens causing AES show seasonal patterns. Herpes simplex virus 1 (HSV-1) another common cause of viral CNS infections in adults in Vietnam, shows no evidence of seasonality [51] with most cases occurring when the virus which is typically acquired during childhood, reactivates [52]. It should also be noted that not all causes of AES are infectious. Studies conducted in Bac Giang province in northern Vietnam found a spatio-temporal association between the incidence of AES and the harvesting of litchis [53, 54]. Additionally, cases of autoimmune encephalitis have recently been identified in Vietnam [55, 56].

Despite an extensive list of aetiologies of AES, the cause remains unknown in a large percentage of cases with global estimates suggesting this could be as high as 85% [57]. However, this percentage is dependent on the number of pathogens and other causes tested for, and the type of testing undertaken [58] with studies in Vietnam failing to identify an aetiology in 45 to 73% of cases [10–12].

In this study, we aim to understand the spatio-temporal distribution of AES infections in Vietnam and its association with risk factors including climate, landcover, proximity to pigs and JE vaccination coverage. This may help to predict where and when increases in the incidence of AES occur and to make hypotheses about pathogens involved, thus allowing preventative public health measures to be implemented.

## Methods

### Data acquisition and transformation

All covariates were decided a priori based on their potential association with AES based on previous literature.

#### Number of cases of AES, meningitis, dengue fever, ILI, HFMD and S. suis (Table 1)

Forty-two notifiable diseases are reported to The General Department for Preventive Medicine (GDPM), a department within the Ministry of Health, Vietnam, on a daily, weekly or monthly basis depending on their categorisation. The case definitions of each of the notifiable disease are provided in Table S1, supplementary data [1, 59]. The number of cases includes those which are defined as suspected, probable or confirmed based on clinical and laboratory diagnoses. If cases classified as VE are later found to have another notifiable disease such as scrub typhus or West Nile Fever, they will be reclassified. Therefore, in addition to monthly cases of AES (classified as ‘VE’) cases of meningitis, dengue fever and ILI from 1998–2016 and *S. suis* and HFMD from 2011–2016 were obtained for each province. Population data from the General Statistical Office of Vietnam (GSO) were used to calculate the per capita incidence [60].

The provincial boundaries have changed on a repeated basis with a total of 60 provinces as defined in 1998, 61 from 1999–2003, 64 from 2004–2007, and 63 from 2008–2016. Therefore, for consistency, the boundaries as defined in 1998 were used as a reference (Figure S1, supplementary data).

The number of cases of meningitis, dengue fever, ILI, HFMD and *S. suis* were converted to incidence per 100,000 population to improve model fitting.

### ***Age and gender (***Table 1***)***

**Table 1:** The datasets used, their unit of time and source and the resolution of inclusion in the analysis

Dataset	Temporal resolution	Spatial resolution	Source	Time range
Number of cases of AES, meningitis, dengue, ILI, HFMD and *S. suis*	Month	Province	GDPM	1998–2016
Human population	Year	Province	GSO	1998–2016
Human population density	Year	Pixel (0.0008°)	WorldPop	2009
Age	Year	Province	World Pop	2000–2016
Gender	Year	Province	World Pop	2000–2016
Climate	Month	Weather stations	Vietnam Institute of Meteorology, Hydrology and Climate Change (IMHEN)	1998–2016
NDVI	Month	Pixel (0.050°)	NOAA	1998–2016
Elevation	–	Pixel (0.0008°)	CGIAR-CSI	2018
Number of pigs	Year	Province	GSO	1998–2016
Number of hospitals	Year	Province	GSO	2005–2016
Poverty rate	Year	Province	GSO	2006, 2008, 2010, 2012–2016
JE vaccination coverage	Year	Province	NIHE	1998–2016

Age and gender were extracted from 3 arc-resolution “unconstrained” raster files from WorldPop for the years 2000–2016 [61]. Age classes in this dataset are < 1, 1–4, 5–9, 10–14, 15–19, 20–24, 25–29, 30–34, 35–39, 40–44, 45–49, 50–54, 55–59, 60–64, 65–69, 70–74, 75–79, and 80 years and over. The values of the pixels were summed within each provinces’ polygons.

Missing values were imputed using the R package ‘mice’ using the default number of imputations of 5 and the default method of predictive mean matching [62]. The data were extracted using the function complete and action ‘long’ from which the mean of each imputation was calculated for the province and year. The population data obtained from the Vietnamese census in 2019 and WorldPop for the same year by province, age category (aged 15 years and over and aged under 15 years) and gender were compared to determine the quality of the WorldPop data. Figure S2, supplementary data shows the correlation between the two datasets.

### ***Climate (***Table 1***)***

Climatic data were obtained from sixty-seven weather stations situated across the country. These included monthly means for the daily mean minimum and mean maximum temperature (°C), daily means of absolute (g/L) and relative (%) humidities; as well as monthly total precipitation (mm) and cumulative hours of sunshine [63]. Kriging was used to interpolate the values of the climatic variables on a 9,960-cell regular grid (i.e. 0.01 degree resolution) using the function autoKrige() from the R package ‘automap’ which automatically tunes the hyperparameters of the variogram by cross-validation [64]. The interpolated values were then aggregated in the polygons of each province by computing a mean weighted by the province local population relative density defined on the same 9,960-cell grid. Thus, for any given province k, the aggregated value of a climatic variable C reads

$${C}_{k}=\sum_{i=1}^{{n}_{k}}{w}_{k,i}\times {C}_{k, i}$$

where *n*_*k*_ is the total number of cells of the grid within the polygon of the province k, *c*_*k,i*_ is the value of the climatic variable in cell *i* of this province k, and *w*_*k,i*_ is the population weight is the same cell, computed as

$${w}_{k, i}=\frac{{p}_{k, i}}{{\sum }_{j=1}^{{n}_{k}}{P}_{k, j}}$$

where *p*_*k,i*_ is the population size in cell *i* obtained from the WorldPop project 2009 raster file (1/1200 degree resolution) [65].

### ***Normalised difference vegetation index (NDVI) (***Table 1***)***

NDVI provides an estimation of the biomass of photosynthetically active vegetation calculated from the reflected visible and near-infrared light. The higher the NDVI, the higher the amount of biomass [66]. Landcover covariates were found to be influential in a predictive model of the spatial distribution of *Cx. tritaeniorhynchus* developed by Longbottom et al., 2017 [19].

In our study, the monthly NDVI for each province was extracted from raster files obtained from the National Centers for Environmental Information (NOAA) [67] at a resolution of 1/20 degrees and weighted by population density as explained above.

### ***Elevation (***Table 1***)***

Elevation was also shown to be influential in a model used to predict the distribution of *Cx. tritaeniorhynchus* [19]. In our study, elevation data were provided by the National Aeronautical Space Administration (NASA) Shuttle Radar Topographic Mission (SRTM) digital elevation models (DEM). These were obtained from the Consortium of International Agricultural Research Centres Consortium for Spatial Information (CGIAR-CSI) GeoPortal [68] at a resolution of 1/1200 degrees. All elevation data were weighted by population density as explained above.

### ***JEV vaccination coverage (***Table 1***)***

Vaccination against Japanese encephalitis virus is part of the Expanded Programme on Immunization (EPI) with children aged 1–5 years receiving two doses of vaccine 1–2 weeks apart. This is followed by a booster dose after a year. The vaccination programme commenced in 1997 only in high-risk area [2] with campaign immunization. This expanded to all provinces by the end of 2014 and from 2015, the JEV vaccine was introduced in routine immunization for most provinces in Vietnam. Yearly data of vaccination coverage by province were provided by the National Institute of Hygiene and Epidemiology (NIHE). The variable for JEV vaccination coverage in a given province was calculated as

$${P}_{t}=\frac{\sum_{i=1998}^{t}{v}_{i}}{{\sum }_{i=1998}^{t}\;{p}_{i}}$$

where *v*_*i*_ is equal to the number of people vaccinated with three doses at time i; *p*_*i*_ is the target population for vaccination at time i; and *t*, the time.

### ***Number of pigs and hospitals and poverty rate (***Table 1***)***

The number of pigs and hospitals and poverty rate per province were obtained from the GSO for each year. Missing values were not random and existed for the years 1998–2004 for the number of hospitals and 1998–2005, 2007, 2009 and 2011 for the poverty rate. Missing values were imputed using the R package ‘mice’ using the default number of imputations of 5 [62]. The data were extracted using the function complete and action ‘long’ from which the mean of each imputation was calculated for the province and year. The number of pigs per 100,000 human population provided an estimate of the proximity of pigs to people, hospitals per 100 km^2^ as a proxy for access to healthcare, and poverty as a potential confounder in the models. The covariate for the pigs per 100,000 population was standardized to improve model fitting by subtracting the mean of the covariate from the covariate and dividing by the standard deviation of the covariate.

### Data cleaning

All analyses and data cleaning were conducted using R statistical programming software version 3.6.1 [69].

Outliers and erroneous values from continuous data obtained from interpolation, or from the raster files were identified using the R package ‘anomalize.’ ‘anomalize’ decomposes the time series for each province and detects anomalies amongst the residuals using both the generalized extreme studentized deviation and inter-quartile range (IQR) [70]. The outliers and erroneous values were replaced with the mean of the upper and lower recomposed values which were generated from the upper and lower limits of the anomalies (the ‘remainders’) and the season and trend from the decomposition of the time series (Table S2, supplementary data). Missing data for NDVI for all provinces in November 1998 and September 2005 and for four provinces in March 1998 and two provinces in March 2005 were unable to be corrected using this method and therefore an average of the values from the same month recorded during the remaining eighteen years for each corresponding province were used. A high outlier of 242 cases (69.1 per 100,000 population) of AES in Kon Tum in January 2002 was removed as was presumed to be a data entry error.

### Analysis

A descriptive analysis of the spatio-temporal incidence of AES and the climatic covariates was initially performed. This was followed by the identification of spatial clusters of the mean annual incidence of AES from 1998 until 2016 between provinces. The local indicators of spatial associations (LISA) was used to define clusters of high and low incidence of AES using the package ‘rgeoda’ [71]. The spatial weights were calculated using the ‘Queen contiguity weights’ function which assumes that the spatial units (the provinces) share a common border. The local Moran statistic with a significance cut-off of 0.05 and 999 permutations, was used to determine spatial clusters and outliers between the provinces. Spatial clusters are defined as those with positive spatial correlation e.g. High-High incidence of AES or Low-Low incidence of AES and spatial outliers as those with negative spatial correlation e.g. High-Low or Low–High [72]. Finally, multivariable models were constructed.

Given the potential for correlation between covariates [73] Pearson’s correlation coefficient was used to determine collinearity among the climatic covariates. The covariates with a correlation coefficient of 0.90 or greater including maximum temperature, minimum temperature and absolute humidity (Table S2, supplementary data) were not included in the same models. Additionally, as surveillance for HFMD and *S. suis* commenced later and data were only available from 2011, these were included in different models. Lags of 0, 1 or 2 months were considered for the climatic covariates and NDVI, to account for delays between conditions suitable for the breeding and development of vectors and the reporting of cases. An additional lag of 3 months was included for rainfall as stagnant water may remain for a prolonged period. Annual harmonic terms sin(2*$\pi$*month/12) and cos(2*$\pi$*month/12) were included to account for seasonality. The covariate for age was given as the ratio between the proportion of those less than 15 years (children) and for gender, the proportion of males. Maximum temperature, minimum temperature and absolute humidity were all highly correlated with a Pearson correlation coefficient of greater than 0.9 (Table S2, supplementary data) resulting in the fitting of six separate models using data from 1998 to 2016 both with and without meningitis, dengue and ILI included as covariates, and three models using data from 2011 to 2016 with meningitis, dengue, ILI, *S. suis* and HFMD included as covariates. A total of nine models were therefore constructed based on different combinations of these covariates fitted with some random effects to account for potential spatio-temporal autocorrelation as detailed below (Table S3, supplementary data).

The search for the most appropriate modelling framework for our data and questions under investigation was performed as follows:

1. Poisson linear mixed-effect models (LMMs) were initially fitted as the simplest framework for each of the six models described above with province, year and month fitted as random effects and log of the population as an offset. All models were checked for overdispersion within the data using the function dispersiontest() from the package ‘AER’. This function tests the null hypothesis of equidispersion against over or underdispersion within the model [74].
2. In the event of overdispersion, the Poisson LMMs would be rejected and negative binomial linear mixed-effect models fitted using the package ‘MASS’ [75] with province, year and month fitted as random effects and log of the population as an offset. Spatial autocorrelation of the residuals of the models was determined using the moran.mc() function, a permutation test for Moran’s I statistic using 1000 simulations from the package ‘spdep’ [76]. Temporal autocorrelation of the residuals of the models was determined using the Box.test() function to compute the Ljung—Box test statistic for independence in a time series (the null hypothesis) [77].
3. In the event of spatial and/or temporal autocorrelation left among the residuals, the negative binomial LMMs would be rejected and spatio-temporal negative binomial LMMs fitted under the Bayesian approach using the inla() function from the package ‘INLA’ [78]. The closest neighbouring province would be added as random effect using the f() function with a Besag-York-Mollie (bym) model [79] and year and month added as random effects using the Random Walk of order 1 model. Alternatively, province, year and month would be added as random effects with no defined model. The log of the population would be added as an offset. The Watanabe-Akaike and deviance information criterion (WAIC and DIC, respectively) would be used to assess the fit of the models.

## Results

**Fig. 1:**
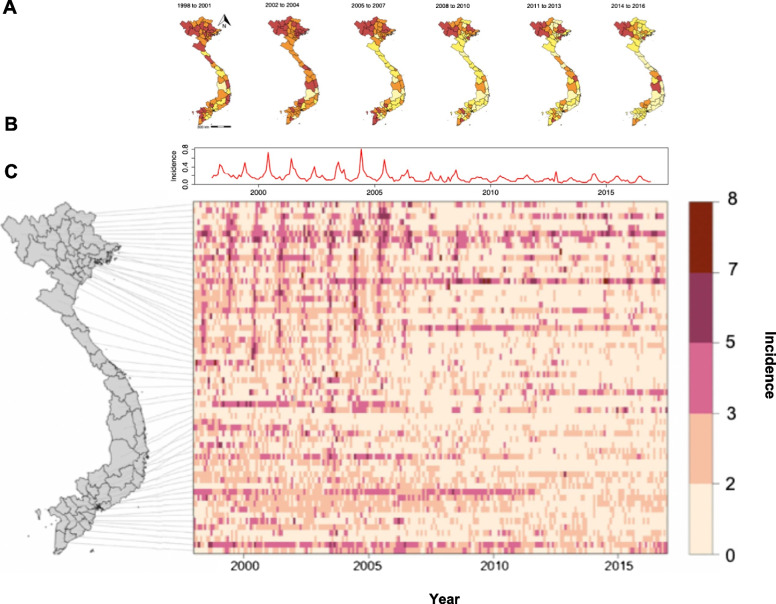
**A** Choropleth maps of the mean annual incidence of AES by province during different periods; **B** Time series of the national incidence of AES per month; **C** Heatmap of the incidence per 100,000 population of AES by province for each month included in the analysis. Incidence refers to the number of cases of AES per 100,000 population. In C) each row represents a province ordered by the latitude of its geographical centroid. The provinces in the heatmap correspond to those in the map of Vietnam on the left. The columns represent each month (1:216) from January 1998 to December 2016

There was a 63.3% decline in the national incidence of AES from 3.0 cases per 100,000 population in 1998 to 1.1 cases per 100,000 population in 2016 with the highest monthly incidence in June 2004 (0.8 cases per 100,000 population). However, in eleven of the sixty provinces, an increase in incidence was seen over time with the highest absolute increase seen in Son La (5.7 to 11.3 cases per 100,000 population (98.2%,)) and Lao Cai (0.9 to 5.1 cases per 100,000 population (466.7%)), both in the Northwest region. The mean annual incidence of AES was highest in Son La and Lai Chau provinces in the Northwest region (9.3 cases per 100,000 population (standard deviation (SD) = 5.1) and 8.4 cases per 100,000 population (SD = 3.7), respectively) and lowest in Phu Yen in the South Central Coast region and Dong Nai in the Southeast region (0.2 cases per 100,000 population (SD = 0.2) and 0.3 cases per 100,000 population (SD = 0.4), respectively). The mean national monthly incidence was highest in June (0.4 cases per 100,000 population, SD = 0.2) and lowest in January (8.1 × 10^–2^ cases per 100,000 population, SD = 3.6 × 10^–2^) (Fig. 1).

**Fig. 2:**
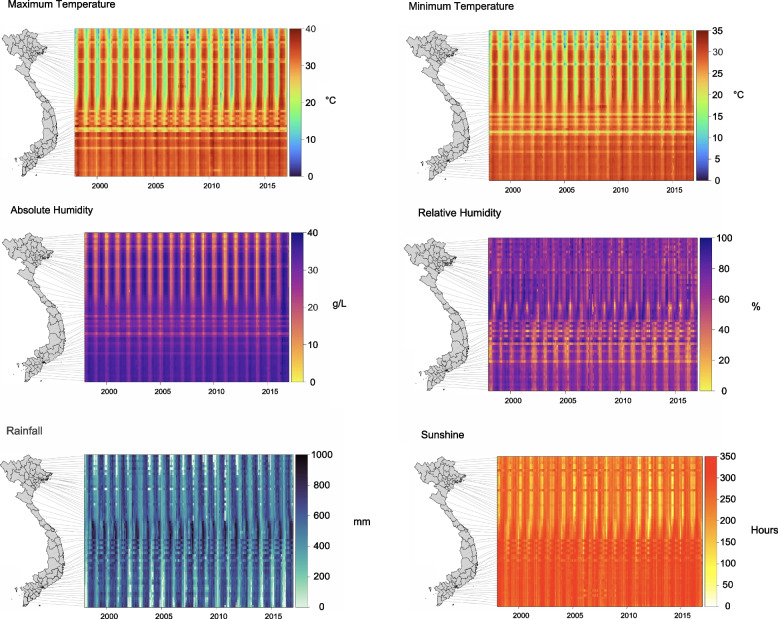
Heatmaps of the monthly interpolated climatic covariates weighted by population density and replacement of the outliers by province. Each row represents a province ordered by the latitude of its geographical centroid. The provinces in the heatmap correspond to those in the map of Vietnam on the left. The columns represent each month (1:216) from January 1998 to December 2016

The climate differed by region. Provinces in southern Vietnam showed a high temperature and high absolute humidity throughout the year. However, the magnitude of seasonality of both of these climatic covariates increased with latitude with cooler, drier winters and warmer, humid summers more apparent in the northern provinces. The provinces in the Central Highlands region were an exception to this trend, where the temperatures and absolute humidity were lower throughout the year due to their higher elevation. The strength of the seasonality of relative humidity appeared less noticeable in the provinces at higher latitude with a greater effect seen in central and southern provinces. Rainfall showed seasonality throughout the country, with different timing in the north, center and south and with the highest rainfall recorded in the South Central Coast. The hours of sunshine were lowest during the winter in the northern provinces and the summer in the southern provinces. The minimum hours of sunshine in the northern provinces were almost equal to the maximum hours of sunshine in the southern provinces (Fig. 2).

**Fig. 3:**
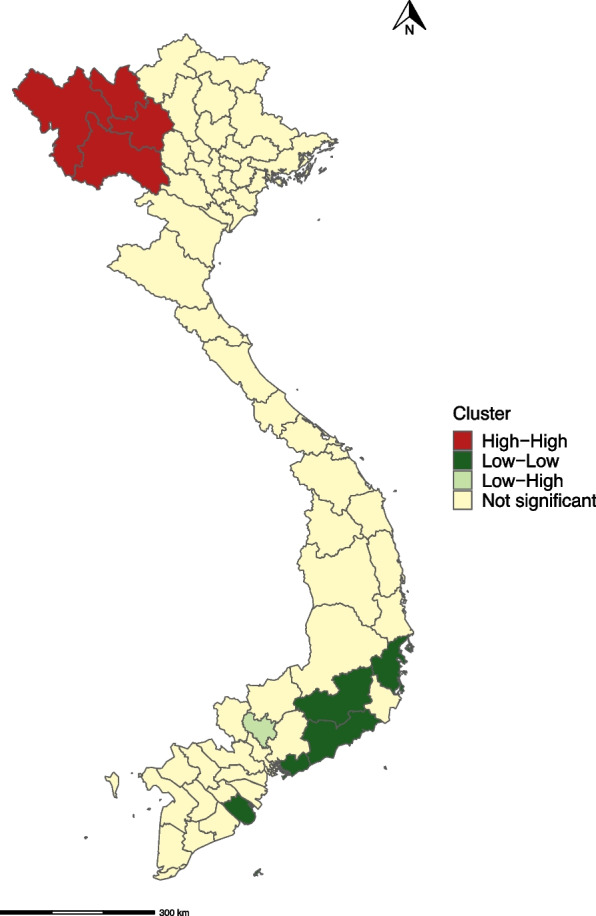
Local indicators of spatial association (LISA) cluster map of the mean annual incidence of AES from 1998 to 2016

A cluster of high-high incidence of AES over the study period included the provinces of Lai Chau, Son La, Lao Cai and Yen Bai in Northwest Vietnam. A cluster of low-low incidence of AES was seen in Southeast Vietnam and the South Central Coast including the provinces of Tra Vinh, Binh Thuan and Lam Dong (Fig. 3).

### Model framework selection

All nine Poisson LMMs showed evidence of overdispersion with alpha values ranging from 5.32 (model 5) to 8.01 (model 9), all with *p* values of < 0.001 and were therefore rejected (Tables S4 and S5, supplementary data). All nine negative binomial LMMs showed evidence of spatial autocorrelation of the residuals with the Moran’s I statistic giving a *p* value of < 0.001 (Tables S6 and S7, supplementary data). However, two of the negative binomial LMMs showed evidence of temporal autocorrelation among the residuals with the Ljung—Box test statistic giving *p* values greater than 0.05 (Tables S6 and S8, supplementary data). The negative binomial LMMs were thus rejected and therefore Spatio-temporal negative binomial LMMs were fitted as the final models. Among the Spatio-temporal models using data from 1998 to 2016 inclusive of meningitis, dengue and ILI as covariates [1–3], model 1 had the lowest WAIC and DIC (44,239.37 and 43,509.41, respectively), among the models using data from 2011 to 2015 [4–6], model 4 had the lowest WAIC (44,224.01) and model 5, the DIC (43,485.15) and among the models using data from 1998 to 2016 but excluding meningitis, dengue and ILI [7–9], model 8 had the lowest WAIC (44,491.43) and model 7, the lowest DIC (43,654.6) (Table S9, supplementary data). Therefore, 1, 4 and 8 were selected as the final models.

### Results from the final spatio-temporal negative binomial models

**Fig. 4:**
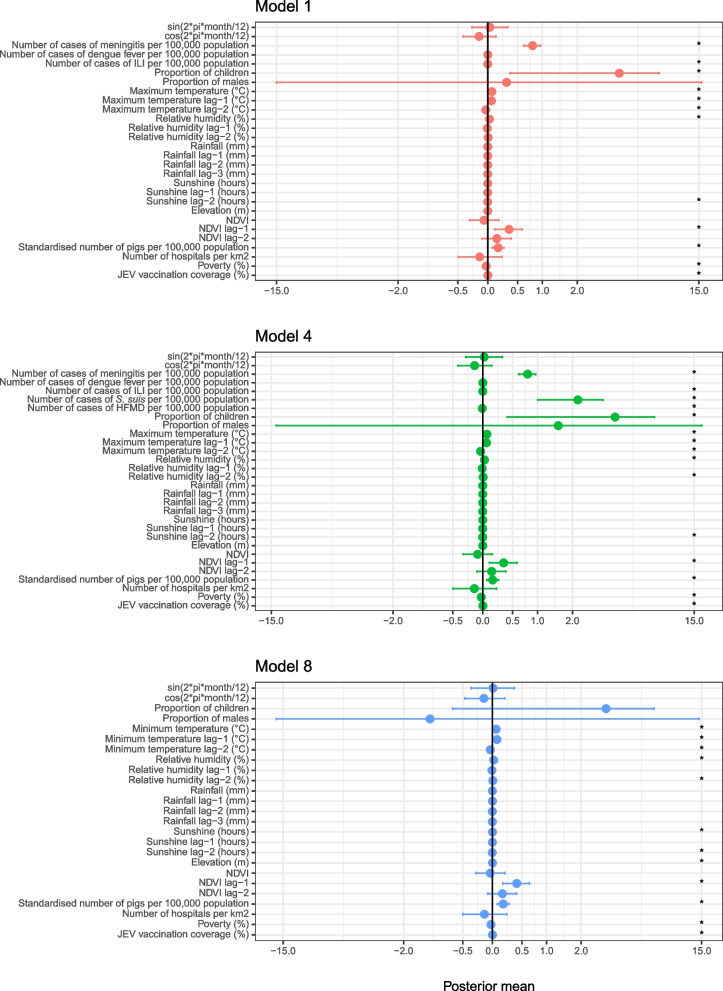
The mean posterior estimates of the spatio-temporal negative binomial models 1, 4 and 8. The dots show the posterior mean and the error bars, the 95% CI. The presence of the asterix (*) is given when the 95% CI does not cross zero

In both of the spatio-temporal negative binomial models 1 and 4, the incidence of meningitis and ILI per 100,000 population was positively associated with the number of cases of AES with the highest posterior mean in model 1 for meningitis (7.87 × 10^–1^ (95% credible interval (CI) 6.14 × 10^–1^-9.60 × 10^–1^)) and ILI (4.93 × 10^–4^ (95% CI 2.51 × 10^–4^-7.34 × 10^–4^)). In model 4, there was a positive association between the number of cases of AES and the incidence of *S. suis* (2.20 (95% CI 1.01-3.40)) and a negative association with the incidence of HFMD (-6.58 × 10^–3^ (95% CI -1.13 × 10^–2^-(-1.92 × 10^–3^))). There was no association with the incidence of dengue. The maximum temperature with no lag and with a lag of one month were positively associated with the number of cases of AES in models 1 and 4 with the highest posterior mean with no lag and a lag of one month both in model 4 (6.67 × 10^–2^ (95% CI 4.04 × 10^–2^-9.30 × 10^–2^) and 6.08 × 10^–2^ (95% CI 3.16 × 10^–2^ - 9.00 × 10^-2^), respectively). However, maximum temperature with a lag of two months was negatively associated in both models with model 4 having the lowest posterior mean (6.67 × 10^–2^ (95% CI 4.04 × 10^–2^ - 9.30 x × 10^–2^)). In model 8, minimum temperature with no lag and a lag of one month were both positively associated with the number of cases of AES (6.14 × 10^–2^ (95%CI 3.56 × 10^–2^- 8.74 x 10^-2^) and 7.39 x 10^-2^ (95%CI 4.33 x 10^-2^ - 1.04 x 10^-1^), respectively). Minimum temperature with a lag of two months months was negatively associated with the number of cases of AES in model 8 (-3.35 × 10^–2^ (95% CI -5.86 × 10^–2^-(-8.47 × 10^–3^))). A positive association with relative humidity with no lag was evident in all models, with model 4 having the highest posterior mean (2.80 × 10^–2^ (95% CI 1.55 × 10^–2^-4.06 × 10^–2^)) and with a lag of two months in models 4 and 8, with 4 having the highest posterior mean (7.12 x 10^-3^ (95%CI 3.38 x 10^-4^- 1.39 x 10^-2^)). No association was seen with relative humidity with a lag of one month. A positive association with sunshine with no lag was also evident in model 8 (1.59 × 10^–3^ (95% CI 5.86 × 10^–4^-2.59 × 10^–3^)) however, no association was seen with a lag of one month and a lag of two months was negatively associated in all three models with model 8 giving the lowest posterior mean (-2.39 × 10^–3^ (95% CI -3.35 × 10^–3^ – (-1.42 × 10^–3^)). There was no association between rainfall and AES. A positive association with NDVI with a lag of one month was evident in all models, with model 8 having the highest posterior mean (4.06 × 10^–1^ (95% CI 1.70 × 10^–1^-6.42 × 10^–1^)). The was no association between AES and NDVI with no lag and a lag of two months. Elevation was positively associated with the number of cases of AES in model 8 (1.45 × 10^–3^ (95% CI 3.33 × 10^–4^ to 2.57 × 10^–3^)) (Fig. 4 and Table S10, supplementary data).

The standardized number of pigs per 100,000 human population was positively associated with AES in all models, the highest seen in model 8 (1.76 × 10^–1^ (95% CI 7.83 × 10^–2^-2.73 × 10^–1^)). JE vaccination coverage was also positively associated with AES in all models with the highest posterior mean in model 4 (2.50 × 10^–3^ (95%CI 1.39 × 10^–3^–3.61 × 10^–3^)). Poverty was negatively associated with AES in all three models with the lowest posterior mean in model 1 (-2.48 × 10^–2^ (95%CI -3.53 × 10^–2^-(-1.43 × 10^–2^))). The proportion of children was positively associated in models 1 and 4, with model 4 having the highest posterior mean (4.15 (95% CI 4.04 × 10^–1^ – 7.88)). There was no association with gender or the number of hospitals per 100 km^2^ (Fig. 4 and Table S10, supplementary data).

## Discussion

This study of the spatio-temporal incidence of Vietnam from 1998 to 2016 showed that the majority of cases of AES occurred in the summer months in northern Vietnam and that number of cases of AES is positively associated with climatic and landcover covariates including temperature, relative and absolute humidity and sunshine and NDVI after adjusting for potential confounders. Additionally, there was evidence of high-high clustering in the provinces in northwest Vietnam. The descriptive analysis of our study builds and correspond with that conducted by Yen et al., 2010 [2]. However, while Yen et al., 2010 included cases from 1998–2007, our study included those until 2015 [2]. It was important to analyse more recent data as the JEV vaccination programme did not reach all provinces until 2015. Furthermore, Vietnam has seen significant recent economic growth over this time, and was reclassified from low to lower middle-income country by 2011. As a result of this, the country likely experienced an improvement in public health, and change in agricultural practices which may have affected the abundance of vectors and contributed to the decline in AES over time seen in our study. Additionally, the work of Yen et al., 2010 focused on the descriptive epidemiology of AES compared to our work, which included multivariable spatio-temporal modelling [2].

The spatio-temporal negative binomial models used in our study demonstrated that temperature had the strongest correlation and sunshine, the least. A similar finding was seen in the study by Lee et al., 2017 conducted from 2004 to 2013 in Son La and Thai Binh provinces however, the authors also documented a positive association with rainfall [8]. This analysis was however, limited to two provinces in northern Vietnam which may explain these differences and did not include temperature and rainfall in the same models due to collinearity between the covariates [8]. While Lee et al., 2017 discuss the potential for sunshine to promote the breeding of *Culex* mosquitoes and highlight other studies which showed a positive association between sunshine and cases of JE [8], given the positive correlation between TB and sunshine in the paper by Bonell et al., 2020, we might also consider TBM as a cause of AES [61]. However, interestingly in our study we saw a negative association between both sunshine and a minimum temperature at a lag of two months and the number of cases of AES. This may suggest that too high a temperature or too much sunlight, may negatively impact the early development of some forms of mosquito larvae.

The association with temperature and humidity corresponds with the epidemiology of vector-borne diseases. JEV, the most common aetiology of viral encephalitis in Vietnam, has been shown to be positively associated with temperature and humidity in studies in Taiwan, Nepal and India, including with lags of one to two months [72, 80–82]. However, the association with rainfall was more varied with a positive association seen in one study in China [80] and a negative association in a separate study in the same country [83]. This emphasises the fine balance between the provision of sufficient water to allow a breeding ground for mosquito larvae, and flooding which washes these away. In Vietnam, temperature, humidity and rainfall have also shown a positive association with the incidence of dengue [34, 84]. However, in Thailand, the majority of cases occurred within a mean temperature of 27–29.5 °C corresponding with range providing the optimal extrinsic incubation period (EIP) of the virus and survival of the adult mosquito [85]. Although we may expect to see a reduction in the number of competent vectors at higher elevations, there is evidence that climate change is allowing these to survive [86, 87]. Therefore, it was not necessarily surprising that even after adjusting for climatic covariates in the INLA models, elevation was positively associated with the number of cases of AES in two of the three final models, particularly as the highest mean elevation per province in Vietnam was just over 1000 m in Lam Dong province, in the Central Highlands.

In addition to JE and dengue fever, other vector-borne diseases have similar epidemiological associations with climate. In the neighbouring countries Laos and Cambodia, *Orientia tsutsugamushi* (scrub typhus) was the second most common cause of CNS infections in children and adults [88, 89]. Headache and altered consciousness were also frequently reported as symptoms in a study of scrub typhus in Hanoi, northern Vietnam [88–90]. *O. tsutsugamushi* is transmitted by the bite of an infected trombiculid mite (chigger) [91]. Like mosquitoes, the chiggers’ optimal development and reproduction is dependent on a temperature which is neither too warn nor too cold and a high relative humidity [91, 92]. As a result of this, the incidence of scrub typhus is seasonal in a number of countries [93–95]. Although rarer, accounting for 1.8% of CNS infections in a study in mainland China [96], tick-borne encephalitis also shows seasonality hence the incidence of tick-borne encephalitis (TBE) shows seasonality [97, 98] due to the sensitivity of the Ixodid ticks which transmit tick-borne encephalitis virus (TBEV) to changes in temperatures and humidity [99].

In our models, NDVI was positively associated with AES at a lag of minus one month. While NDVI has been shown to be positively associated with the number of cases of JEV infection in mainland China [80] and incidence of scrub typhus in Taiwan [100], it was negatively associated with dengue in Taiwan, possibly due to the role of population density in transmission of the virus [101]. The lag of one month demonstrates the time between the provision of suitable landcover for vector breeding and infection of humans with the pathogen.

Looking more closely at the spatio-temporal distribution of AES, we can begin to make hypotheses about the possible aetiologies. The high incidence of cases in provinces in northwest Vietnam and increase in the seasonality of incidence with latitude corresponds with the epidemiological pattern of JE. JE has historically seen seasonal epidemics in northern provinces from May–July and year-round cases in southern Vietnam [102, 103]. Furthermore, the reduction in incidence of AES over time corresponds with the rollout of the JEV vaccination campaign and introduction in the expanded programme for immunization (EPI) [104]. The positive association with JEV vaccination coverage may contradict this hypothesis as we would expect to see a higher incidence of AES in those provinces with a lower vaccination coverage. However, in the latter, it is known that despite high vaccination coverage in provinces in proximity to Laos, such as Son La and Lai Chau, outbreaks of AES still occur with the majority of cases unvaccinated as they did not meet the age criteria required to participate in the immunization campaigns [105], Finally, the positive association between the number of cases of AES and the number of pigs per 100,000 human population may also support the hypothesis that many cases of AES are due to JEV despite proximity to pigs as a risk factor for infection remaining inconclusive [106, 107].

In addition to JEV, *S. suis* shows a similar spatio-temporal pattern to AES [47, 49]. While it was under-recognised as a cause of bacterial meningitis, particularly in northern Vietnam [49], efforts to increase awareness and the availability of CSF and blood culture, will reduce the likelihood of it being misdiagnosed as AES. However, given the positive associations between meningitis, *S. suis* and the number of pigs and AES in our models it is possible that some cases of *S. suis* infection, possibly in lower resourced healthcare centres, are misclassified as AES. While the association between AES and NDVI may suggest a vector-borne aetiology, pigs are more likely to be reared and therefore consumed in rural areas.

Other common causes of CNS infection in Vietnam show a different spatio-temporal distribution to AES. Although influenza virus is not a common cause of CNS infections in Vietnam (there was only one case of encephalitis in children caused by influenza A(H5N1)) [11, 50] and in a study of adults admitted to a hospital in HCMC with CNS infections, none were caused by influenza A or B [10], like AES, it shows an association with absolute humidity. However, unlike AES, cases in northern Vietnam peak in the late summer/autumn. Despite these different spatio-temporal distributions, a positive association was still evident between AES and ILI in the spatio-temporal negative binomial models and therefore influenza virus cannot be excluded as potential cause of AES. However, both dengue and HFMD showed no association with cases of AES in the INLA models. Despite its association with temperature and humidity, with the exception of Hanoi, northern Vietnam sees few cases of dengue, with the majority of cases occurring in the central and southern provinces, where peaks in incidence occur during the rainy season from July to September [34, 108]. Most cases of HFMD occurring in southern Vietnam with peaks in incidence from March to May and September to December [40] and major outbreaks occurring in 2011–2012 and 2018 [109].

By understanding the spatio-temporal distribution of AES and the association with climatic factors, we can help to predict when and where increases in the incidence of cases will be seen. Given the positive association of AES with temperature, humidity, NDVI and number of pigs, and similar spatio-temporal patterns with JE, there is a suggestion that many cases may be due to JEV or other arthropod-borne pathogens for which the vectors are similarly dependent on these factors. The case for JE, is supported by the positive association between AES and the proportion of children aged less than 15 years with a relatively high posterior mean compared to other covariates in two of the final models. JE is predominantly seen in children however, in areas of lower endemicity or where the virus has been more recently introduced, it may be seen in adults who have no pre-existing immunity [13].

As data for cases of JE were only available from sentinel sites these were not included in our models however, we would still suggest using the models to help guide public health measures in the control of JE such as vaccination campaigns. In addition to *S. suis*, it is possible that other diseases such as dengue and scrub typhus may have been misclassified as AES among patients treated in healthcare centres with fewer diagnostics. Although we attempted to adjust for this in our models by including the number of cases due to common AES aetiologies, our models may still have been subject to reporting bias. Similarly, in healthcare settings where there are fewer diagnostics, for example in more rural provinces, patients with pathogens such as dengue virus may have been misclassified as AES unless they were subsequently transferred to a tertiary centre for care. Despite this, we recognise the importance of pathogens other than JEV which are likely to contribute to the aetiology of AES including *S. suis* and *O. tsutsugamushi* and would recommend conducting sentinel site surveillance to test for these. Additionally, given the potential for ecological fallacy as data are provided at the level of province, and reporting biases either due to differences in the interpretation of case definitions, resources used to diagnose AES and selection biases due to differences in access to healthcare, we would recommend conducting a case control study to determine individual risk factors for AES. Given that data on influenza were only available from sentinel sites, we used ILI as a proxy however, this may be subject to misclassification errors as discussed in a similar paper evaluating the association between sunshine and tuberculosis in Vietnam [63]. Finally, despite the inclusion of multiple covariates in the models, we could have added those which may have been more highly correlated with mosquito suitability such as tasselled cap wetness and brightness.

## Conclusions

AES shows spatio-temporal variability in Vietnam and an association with climatic variables, NDVI, elevation, the proportion of children and the number of pigs. This may suggest that a large proportion of undiagnosed cases are due to JEV or other arthropod-borne pathogens which would indicate the need for further vaccination campaigns. However, additional surveillance and improved diagnostics and research are recommended to better understand other potential aetiologies.

## Supplementary Information

**Additional file 1:** **Table S1.** Case definitions for the notifiable diseases included in the analyses. **Figure S1.** Provinces of Vietnam in 1998 by region. **Figure S2.** Correlation between the population from the 2019 Vietnamese census and 2019 data extracted from WorldPop by age category, gender and province. The red line indicates the line of best fit. **Table S2.** Pearson correlation coefficients for climatic and landcover covariates. **Table S3.** Covariates included in the nine different models. **Table S4.** Poisson linear mixed models showing the association between the number of cases of AES and each of the covariates. **Table S5.** Output of the ‘dispersiontest’ function for each of the Poisson linear mixed models. **Table S6.** Negative binomial linear mixed models showing the association between the number of cases of AES and each of the covariates. **Table S7.** Spatial autocorrelation amongst the residuals from the six negative binomial linear mixed models. **Table S8.** Temporal autocorrelation amongst the residuals from the six negative binomial linear mixed models. **Table S9.** The Watanabe-Akaike criterion and deviance information criterion from the final spatio-temporal negative binomial models. **Table S10.** Final spatio-temporal negative binomial models showing the association between the number of cases of AES and each of the covariates.

## Data Availability

Data are available from the following sources. https://github.com/epix-project/gso. https://www.noaa.gov/. https://www.worldpop.org/. https://cgiarcsi.community/. https://github.com/epix-project/imhen. https://github.com/choisy/epiVN. A copy of the programming code and final dataset for analysis can be made available on request to the corresponding author.

## Abbreviations

AES: Acute encephalitis syndrome
bym: Besag-York-Mollie
CGIAR-CSI: Consortium of International Agricultural Research Centres Consortium for Spatial Information
CI: Credible interval
CNS: Central nervous system
CSF: Cerebrospinal fluid
DEM: Digital Elevation Models
DENV: Dengue virus
DIC: Deviance Information Criterion
EPI: Expanded Programme on Immunization
GDPM: General Department for Preventive Medicine
GLMMs: Generalised linear mixed models
GSO: General Statistical Office of Vietnam
HFMD: Hand foot and mouth disease
Hib: *Haemophilus influenzae* Type b
ILI: Influenza-like-illness
IMHEN: Vietnam Institute of Meterology, Hydrology and Climate Change
INLA: Integrated Nested Laplace Approximation
IQR: Inter-quartile range
JE: Japanese encephalitis
JEV: Japanese encephalitis virus
NASA: National Aeronautical Space Administration
NDVI: Normalized Difference Vegetation Index
NIHE: National Institute of Hygiene and Epidemiology
NOAA: National Centers for Environmental Information
PCR: Polymerase chain reaction
RT-PCR: Reverse transcriptase polymerase chain reaction
SD: Standard deviation
SRTM: Shuttle Radar Topographic Mission
TBE: Tick-borne encephalitis
TBEV: Tick-borne encephalitis virus
VE: Viral encephalitis
WAIC: Watanabe-Akaike Information Criterion
