# Supplementary material for: The spatio-temporal distribution of acute encephalitis syndrome and its association with climate and landcover in Vietnam
Source: BMC Infect Dis. 2023 Jun 13;23:403. doi: 10.1186/s12879-023-08300-1 (PMC10262680; doi:10.1186/s12879-023-08300-1)
Supplement: Supplementary file 1 — Additional file 1: Table S1. Case definitions for the notifiable diseases included in the analyses. Figure S1. Provinces of Vietnam in 1998 by region. Figure S2. Correlation between the population from the 2019 Vietnamese census and 2019 data extracted from WorldPop by age category, gender and province. The red line indicates the line of best fit. Table S2. Pearson correlation coefficients for climatic and landcover covariates. Table S3. Covariates included in the nine different models. Table S4. Poisson linear mixed models showing the association between the number of cases of AES and each of the covariates. Table S5. Output of the ‘dispersiontest’ function for each of the Poisson linear mixed models. Table S6. Negative binomial linear mixed models showing the association between the number of cases of AES and each of the covariates. Table S7. Spatial autocorrelation amongst the residuals from the six negative binomial linear mixed models. Table S8. Temporal autocorrelation amongst the residuals from the six negative binomial linear mixed models. Table S9. The Watanabe-Akaike criterion and deviance information criterion from the final spatio-temporal negative binomial models. Table S10. Final spatio-temporal negative binomial models showing the association between the number of cases of AES and each of the covariates. [file 12879_2023_8300_MOESM1_ESM.doc]

**Supplementary data**

Table S1. Case definitions for the notifiable diseases included in the analyses

| **Notifiable disease** | **Definition (suspected case)** | **Definition (possible or probable case)** | **Definition (confirmed case)** |
| --- | --- | --- | --- |
| Viral encephalitis | A case with signs of acute encephalitis - meningitis with such symptoms as sudden high fever, headache, nausea, vomiting, stiff neck, convulsions, paralysis, consciousness disorders (stupor, lethargy, drowsiness, coma). |  | A suspected case having test results positive for the following viruses:  -JE: Cerebrospinal fluid (CSF) testing positive for specific IgM antibodies or detection of specific gene segments of the virus by molecular biological techniques.  -Encephalitis due to other viruses (Herpes virus, intestinal/enteric virus): CSF tests to identify specific gene segments of the virus using molecular biological techniques. |
| Meningitis | - Fever  - Inflammation of the upper respiratory tract (sore throat, cough, runny nose)  - Headache, nausea, vomiting, stiff neck (infants have bulging fontanels), lethargy, sensitivity to light.  - If accompanied by sepsis, the patient will appear to have a death rash or show septic shock | A suspected case accompanied by:  - Staying in/going to/from an endemic area within 14 days prior to onset.  And/or  Close contact with a confirmed case within 14 days prior to onset | Meningococcal bacteria are suspected or possible and meningococcal bacteria are identified by one of the following techniques:  - Isolation culture.  - Molecular biology. |
| Dengue fever | A high fever above 38°C for 2-7 days; headache, muscle and joint pain, periorbital pain, congestion, skin rash, signs of bleeding or signs of shock. |  |  |
| ILI | - Fever.  - Headache, muscle pain, fatigue.  - Inflammation of the respiratory tract: runny nose, stuffy nose, sore throat, cough.  - May be accompanied by gastrointestinal symptoms (nausea, vomiting, diarrhea), especially in children. |  | A suspected illness or a case of illness that can be accompanied by a positive test for influenza virus by one of the following tests:  - Rapid antigen detection test.  - Molecular biology method.  - Virus isolation culture. |
| HFMD | Fever and skin rash are presented. The rash is mainly in the form of vesicles mostly found on palms, soles of feet, knees, buttocks, mouth, possibly accompanied by mouth ulcers. | - A suspected case which is epidemiologically linked to a confirmed case, or  - A suspected case and accompanied by one or more complications: myocarditis, acute pulmonary edema, neurological complications. | A suspected case and confirmation of virus causing HFMD in throat swabs or stool or vesicle fluid using reverse transcriptase polymerase chain reaction (RT-PCR. |
| *S. suis* | The patients with meningitis and/or septicemia, possible septic shock accompanied by one or more manifestations of:  - Purpura, petechiae, ecchymoses.  - Hearing loss or deafness.  - Having a history of direct contact with pigs; slaughtering infected or dead pigs; or handling products such as uncooked blood, meat, innards/organs of pigs, within 14 days. |  | Suspect and confirmed cases with detection of *S.suis* in CSF specimen and/or peripheral blood of patients with culture isolation or polymerase chain reaction (PCR). |

Figure S1. Provinces of Vietnam in 1998 by region.

Figure S2. Correlation between the population from the 2019 Vietnamese census and 2019 data extracted from WorldPop by age category, gender and province. The red line indicates the line of best fit.

Table S2. Pearson correlation coefficients for climatic and landcover covariates.

|  | **Maximum temperature (°C)** | **Minimum temperature (°C)** | **Absolute humidity (g/m^3^)** | **Relative humidity (%)** | **Rainfall (mm)** | **Sunshine (hours)** | **NDVI** |
| --- | --- | --- | --- | --- | --- | --- | --- |
| **Maximum temperature (°C)** |  | 0.93 | 0.90 | -0.22 | 0.30 | 0.73 | 0.24 |
| **Minimum temperature (°C)** | 0.93 |  | 0.97 | -0.07 | 0.40 | 0.56 | 0.11 |
| **Absolute humidity (g/m^3^)** | 0.90 | 0.97 |  | 0.06 | 0.48 | 0.50 | 0.10 |
| **Relative humidity (%)** | -0.22 | -0.07 | 0.06 |  | 0.39 | -0.53 | -0.18 |
| **Rainfall (mm)** | 0.30 | 0.40 | 0.48 | 0.39 |  | -0.05 | -0.05 |
| **Sunshine (hours)** | 0.73 | 0.56 | 0.50 | -0.53 | -0.05 |  | 0.32 |
| **NDVI** | 0.24 | 0.11 | 0.10 | -0.18 | -0.05 | 0.32 |  |

Table S3. Covariates included in the nine different models.

| **Covariates** | **Fixed or random effect** | **Model** | | | | | | | | |
| --- | --- | --- | --- | --- | --- | --- | --- | --- | --- | --- |
|  |  | **1** | **2** | **3** | **4** | **5** | **6** | **7** | **8** | **9** |
| sin(2*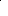*month/12) | Fixed | x | x | x | x | x | x | x | x | x |
| cos(2*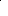*month/12) | Fixed | x | x | x | x | x | x | x | x | x |
| Incidence of meningitis per 100,000 population | Fixed | x | x | x | x | x | x |  |  |  |
| Incidence of dengue fever per 100,000 population | Fixed | x | x | x | x | x | x |  |  |  |
| Incidence of ILI per 100,000 population | Fixed | x | x | x | x | x | x |  |  |  |
| Incidence of HFMD per 100,000 population | Fixed |  |  |  | x | x | x |  |  |  |
| Incidence of *S. suis* per 100,000 population | Fixed |  |  |  | x | x | x |  |  |  |
| Maximum temperature (°C) | Fixed | x |  |  | x |  |  | x |  |  |
| Maximum temperature at a lag of minus one month (°C) | Fixed | x |  |  | x |  |  | x |  |  |
| Maximum temperature at a lag of minus two months (°C) | Fixed | x |  |  | x |  |  | x |  |  |
| Minimum temperature (°C) | Fixed |  | x |  |  | x |  |  | x |  |
| Minimum temperature at a lag of minus one month (°C) | Fixed |  | x |  |  | x |  |  | x |  |
| Minimum temperature at a lag of minus two months (°C) | Fixed |  | x |  |  | x |  |  | x |  |
| Relative humidity (%) | Fixed | x | x | x | x | x | x | x | x | x |
| Relative humidity at a lag of minus one month (%) | Fixed | x | x | x | x | x | x | x | x | x |
| Relative humidity at a lag of minus two months (%) | Fixed | x | x | x | x | x | x | x | x | x |
| Absolute humidity (g/m^3^) | Fixed |  |  | x |  | x |  |  |  | x |
| Absolute humidity at a lag of minus one month (g/m^3^) | Fixed |  |  | x |  | x |  |  |  | x |
| Absolute humidity at a lag of minus two months (g/m^3^) | Fixed |  |  | x |  | x |  |  |  | x |
| Rainfall (mm) | Fixed | x | x | x | x | x | x | x | x | x |
| Rainfall at a lag of minus one month (mm) | Fixed | x | x | x | x | x | x | x | x | x |
| Rainfall at a lag of minus two months (mm) | Fixed | x | x | x | x | x | x | x | x | x |
| Rainfall at a lag of minus three months (mm) | Fixed | x | x | x | x | x | x | x | x | x |
| Sunshine (hours) | Fixed | x | x | x | x | x | x | x | x | x |
| Sunshine at a lag of minus one month (hours) | Fixed | x | x | x | x | x | x | x | x | x |
| Sunshine at a lag of minus two months (hours) | Fixed | x | x | x | x | x | x | x | x | x |
| Elevation (m) | Fixed | x | x | x | x | x | x | x | x | x |
| NDVI | Fixed | x | x | x | x | x | x | x | x | x |
| NDVI at a lag of minus one month | Fixed | x | x | x | x | x | x | x | x | x |
| NDVI at a lag of minus two months | Fixed | x | x | x | x | x | x | x | x | x |
| Proportion of children | Fixed | x | x | x | x | x | x | x | x | x |
| Proportion of males | Fixed | x | x | x | x | x | x | x | x | x |
| Standardised number of pigs per 100,000 human population | Fixed | x | x | x | x | x | x | x | x | x |
| Number of hospitals per 100 km^2^ | Fixed | x | x | x | x | x | x | x | x | x |
| Poverty rate (%) | Fixed | x | x | x | x | x | x | x | x | x |
| JEV vaccination coverage (%) | Fixed | x | x | x | x | x | x | x | x | x |
| Province | Random | x | x | x | x | x | x | x | x | x |
| Month | Random | x | x | x | x | x | x | x | x | x |
| Year | Random | x | x | x | x | x | x | x | x | x |

Table S4. Poisson linear mixed models showing the association between the number of cases of AES and each of the covariates.

| **Model** | **Covariate** | **Estimate** | **Standard error** | **p value** |
| --- | --- | --- | --- | --- |
| 1 | (Intercept) | -1.79 × 10^1 | 9.35 × 10^-1 | <0.001 |
| 1 | sin(2 * pi * month2/12) | 9.88 × 10^-2 | 2.35 × 10^-2 | <0.001 |
| 1 | cos(2 * pi * month2/12) | -2.27 × 10^-1 | 2.16 × 10^-2 | <0.001 |
| 1 | Number of cases of meningitis per 100,000 population | 4.78 × 10^-1 | 2.00 × 10^-2 | <0.001 |
| 1 | Number of cases of dengue fever per 100,000 population | 1.90 × 10^-3 | 5.36 × 10^-4 | <0.001 |
| 1 | Number of cases of ILI per 100,000 population | 4.87 × 10^-4 | 3.48 × 10^-5 | <0.001 |
| 1 | Proportion of children | 4.75 | 2.63 × 10^-1 | <0.001 |
| 1 | Proportion of males | -1.19 × 10^1 | 1.63 | <0.001 |
| 1 | Maximum temperature (°C) | 2.51 × 10^-2 | 6.63 × 10^-3 | <0.001 |
| 1 | Maximum temperature lag-1 (°C) | 7.81 × 10^-2 | 8.27 × 10^-3 | <0.001 |
| 1 | Maximum temperature lag-2 (°C) | 3.27 × 10^-3 | 6.49 × 10^-3 | 0.614 |
| 1 | Relative humidity (%) | 2.56 × 10^-2 | 2.82 × 10^-3 | <0.001 |
| 1 | Relative humidity lag-1 (%) | 1.90 × 10^-2 | 3.16 × 10^-3 | <0.001 |
| 1 | Relative humidity lag-2 (%) | 2.86 × 10^-2 | 2.75 × 10^-3 | <0.001 |
| 1 | Rainfall (mm) | -4.10 × 10^-4 | 9.06 × 10^-5 | <0.001 |
| 1 | Rainfall lag-1 (mm) | 7.11 × 10^-4 | 9.68 × 10^-5 | <0.001 |
| 1 | Rainfall lag-2 (mm) | -6.27 × 10^-4 | 1.02 × 10^-4 | <0.001 |
| 1 | Rainfall lag-3 (mm) | -6.39 × 10^-4 | 1.02 × 10^-4 | <0.001 |
| 1 | Sunshine (hours) | 1.06 × 10^-3 | 2.59 × 10^-4 | <0.001 |
| 1 | Sunshine lag-1 (hours) | 8.69 × 10^-4 | 2.62 × 10^-4 | 0.001 |
| 1 | Sunshine lag-2 (hours) | -1.90 × 10^-3 | 2.62 × 10^-4 | <0.001 |
| 1 | Elevation (m) | 6.25 × 10^-4 | 5.37 × 10^-5 | <0.001 |
| 1 | NDVI | -2.13 × 10^-1 | 5.45 × 10^-2 | <0.001 |
| 1 | NDVI lag-1 | 3.30 × 10^-1 | 5.28 × 10^-2 | <0.001 |
| 1 | NDVI lag-2 | 1.99 × 10^-2 | 5.50 × 10^-2 | 0.718 |
| 1 | Standardised number of pigs per 100,000 population | 2.66 × 10^-1 | 9.06 × 10^-3 | <0.001 |
| 1 | Number of hospitals per 100 km^2^ | 4.71 × 10^-2 | 1.92 × 10^-2 | 0.014 |
| 1 | Poverty (%) | 1.19 × 10^-2 | 7.54 × 10^-4 | <0.001 |
| 1 | JEV vaccination coverage (%) | -4.90 × 10^-3 | 3.72 × 10^-4 | <0.001 |
| 2 | (Intercept) | -1.67 × 10^1 | 9.20 × 10^-1 | <0.001 |
| 2 | sin(2 * pi * month2/12) | 9.23 × 10^-2 | 2.43 × 10^-2 | <0.001 |
| 2 | cos(2 * pi * month2/12) | -2.04 × 10^-1 | 2.23 × 10^-2 | <0.001 |
| 2 | Number of cases of meningitis per 100,000 population | 4.75 × 10^-1 | 2.01 × 10^-2 | <0.001 |
| 2 | Number of cases of dengue fever per 100,000 population | 2.12 × 10^-3 | 5.32 × 10^-4 | <0.001 |
| 2 | Number of cases of ILI per 100,000 population | 5.00 × 10^-4 | 3.46 × 10^-5 | <0.001 |
| 2 | Proportion of children | 4.55 | 2.62 × 10^-1 | <0.001 |
| 2 | Proportion of males | -1.21 × 10^1 | 1.63 | <0.001 |
| 2 | Minimum temperature (°C) | 3.54 × 10^-2 | 6.72 × 10^-3 | <0.001 |
| 2 | Minimum temperature lag-1 (°C) | 6.78 × 10^-2 | 8.20 × 10^-3 | <0.001 |
| 2 | Minimum temperature lag-2 (°C) | -3.53 × 10^-4 | 6.46 × 10^-3 | 0.956 |
| 2 | Relative humidity (%) | 2.35 × 10^-2 | 2.83 × 10^-3 | <0.001 |
| 2 | Relative humidity lag-1 (%) | 1.34 × 10^-2 | 3.15 × 10^-3 | <0.001 |
| 2 | Relative humidity lag-2 (%) | 2.97 × 10^-2 | 2.69 × 10^-3 | <0.001 |
| 2 | Rainfall (mm) | -3.74 × 10^-4 | 8.98 × 10^-5 | <0.001 |
| 2 | Rainfall lag-1 (mm) | 6.78 × 10^-4 | 9.62 × 10^-5 | <0.001 |
| 2 | Rainfall lag-2 (mm) | -6.69 × 10^-4 | 1.03 × 10^-4 | <0.001 |
| 2 | Rainfall lag-3 (mm) | -6.73 × 10^-4 | 1.03 × 10^-4 | <0.001 |
| 2 | Sunshine (hours) | 1.43 × 10^-3 | 2.23 × 10^-4 | <0.001 |
| 2 | Sunshine lag-1 (hours) | 1.88 × 10^-3 | 2.32 × 10^-4 | <0.001 |
| 2 | Sunshine lag-2 (hours) | -1.71 × 10^-3 | 2.28 × 10^-4 | <0.001 |
| 2 | Elevation (m) | 8.04 × 10^-4 | 6.21 × 10^-5 | <0.001 |
| 2 | NDVI | -1.88 × 10^-1 | 5.41 × 10^-2 | 0.001 |
| 2 | NDVI lag-1 | 3.99 × 10^-1 | 5.23 × 10^-2 | <0.001 |
| 2 | NDVI lag-2 | 7.10 × 10^-2 | 5.46 × 10^-2 | 0.194 |
| 2 | Standardised number of pigs per 100,000 population | 2.84 × 10^-1 | 9.05 × 10^-3 | <0.001 |
| 2 | Number of hospitals per 100 km^2^ | 6.62 × 10^-2 | 1.92 × 10^-2 | 0.001 |
| 2 | Poverty (%) | 1.30 × 10^-2 | 7.51 × 10^-4 | <0.001 |
| 2 | JEV vaccination coverage (%) | -5.14 × 10^-3 | 3.75 × 10^-4 | <0.001 |
| 3 | (Intercept) | -1.50 × 10^1 | 9.19 × 10^-1 | <0.001 |
| 3 | sin(2 * pi * month2/12) | 1.25 × 10^-1 | 2.61 × 10^-2 | <0.001 |
| 3 | cos(2 * pi * month2/12) | -1.58 × 10^-1 | 2.32 × 10^-2 | <0.001 |
| 3 | Number of cases of meningitis per 100,000 population | 4.76 × 10^-1 | 2.01 × 10^-2 | <0.001 |
| 3 | Number of cases of dengue fever per 100,000 population | 1.92 × 10^-3 | 5.32 × 10^-4 | <0.001 |
| 3 | Number of cases of ILI per 100,000 population | 4.93 × 10^-4 | 3.49 × 10^-5 | <0.001 |
| 3 | Proportion of children | 4.76 | 2.63 × 10^-1 | <0.001 |
| 3 | Proportion of males | -1.31 × 10^1 | 1.64 | <0.001 |
| 3 | Absolute humidity (g/m3) | 4.12 × 10^-2 | 5.09 × 10^-3 | <0.001 |
| 3 | Absolute humidity lag-1 (g/m3) | 4.01 × 10^-2 | 6.09 × 10^-3 | <0.001 |
| 3 | Absolute humidity lag-2 (g/m3) | 3.01 × 10^-3 | 4.99 × 10^-3 | 0.547 |
| 3 | Relative humidity (%) | 1.67 × 10^-2 | 2.99 × 10^-3 | <0.001 |
| 3 | Relative humidity lag-1 (%) | 8.46 × 10^-3 | 3.34 × 10^-3 | 0.011 |
| 3 | Relative humidity lag-2 (%) | 2.76 × 10^-2 | 2.77 × 10^-3 | <0.001 |
| 3 | Rainfall (mm) | -4.16 × 10^-4 | 9.07 × 10^-5 | <0.001 |
| 3 | Rainfall lag-1 (mm) | 6.02 × 10^-4 | 9.62 × 10^-5 | <0.001 |
| 3 | Rainfall lag-2 (mm) | -6.21 × 10^-4 | 1.03 × 10^-4 | <0.001 |
| 3 | Rainfall lag-3 (mm) | -5.86 × 10^-4 | 1.02 × 10^-4 | <0.001 |
| 3 | Sunshine (hours) | 1.19 × 10^-3 | 2.29 × 10^-4 | <0.001 |
| 3 | Sunshine lag-1 (hours) | 1.76 × 10^-3 | 2.35 × 10^-4 | <0.001 |
| 3 | Sunshine lag-2 (hours) | -1.58 × 10^-3 | 2.36 × 10^-4 | <0.001 |
| 3 | Elevation (m) | 8.40 × 10^-4 | 6.11 × 10^-5 | <0.001 |
| 3 | NDVI | -2.27 × 10^-1 | 5.44 × 10^-2 | <0.001 |
| 3 | NDVI lag-1 | 3.71 × 10^-1 | 5.24 × 10^-2 | <0.001 |
| 3 | NDVI lag-2 | 7.15 × 10^-2 | 5.47 × 10^-2 | 0.191 |
| 3 | Standardised number of pigs per 100,000 population | 2.72 × 10^-1 | 9.00 × 10^-3 | <0.001 |
| 3 | Number of hospitals per 100 km^2^ | 6.47 × 10^-2 | 1.92 × 10^-2 | 0.001 |
| 3 | Poverty (%) | 1.33 × 10^-2 | 7.54 × 10^-4 | <0.001 |
| 3 | JEV vaccination coverage (%) | -5.30 × 10^-3 | 3.77 × 10^-4 | <0.001 |
| 4 | (Intercept) | -2.60 × 10^1 | 1.82 | <0.001 |
| 4 | sin(2 * pi * month2/12) | -8.28 × 10^-2 | 4.23 × 10^-2 | 0.05 |
| 4 | cos(2 * pi * month2/12) | -9.92 × 10^-2 | 3.82 × 10^-2 | 0.009 |
| 4 | Number of cases of meningitis per 100,000 population | 1.36 | 8.07 × 10^-2 | <0.001 |
| 4 | Number of cases of dengue fever per 100,000 population | -5.83 × 10^-4 | 1.07 × 10^-3 | 0.585 |
| 4 | Number of cases of ILI per 100,000 population | 2.39 × 10^-4 | 8.14 × 10^-5 | 0.003 |
| 4 | Number of cases of HFMD per 100,000 population | -1.14 × 10^-2 | 1.46 × 10^-3 | <0.001 |
| 4 | Number of cases of *S. suis* | 1.08 | 1.11 × 10^-1 | <0.001 |
| 4 | Proportion of children | 3.90 × 10^-1 | 6.48 × 10^-1 | 0.548 |
| 4 | Proportion of males | 1.62 × 10^1 | 3.43 | <0.001 |
| 4 | Maximum temperature (°C) | 3.86 × 10^-2 | 1.19 × 10^-2 | 0.001 |
| 4 | Maximum temperature lag-1 (°C) | 7.07 × 10^-2 | 1.48 × 10^-2 | <0.001 |
| 4 | Maximum temperature lag-2 (°C) | -5.19 × 10^-2 | 1.14 × 10^-2 | <0.001 |
| 4 | Relative humidity (%) | 2.12 × 10^-2 | 5.73 × 10^-3 | <0.001 |
| 4 | Relative humidity lag-1 (%) | 1.27 × 10^-2 | 6.29 × 10^-3 | 0.044 |
| 4 | Relative humidity lag-2 (%) | -2.47 × 10^-3 | 5.41 × 10^-3 | 0.648 |
| 4 | Rainfall (mm) | -4.39 × 10^-4 | 1.70 × 10^-4 | 0.01 |
| 4 | Rainfall lag-1 (mm) | 4.73 × 10^-4 | 1.77 × 10^-4 | 0.008 |
| 4 | Rainfall lag-2 (mm) | 8.60 × 10^-4 | 1.76 × 10^-4 | <0.001 |
| 4 | Rainfall lag-3 (mm) | -7.17 × 10^-4 | 1.78 × 10^-4 | <0.001 |
| 4 | Sunshine (hours) | 6.51 × 10^-4 | 4.91 × 10^-4 | 0.185 |
| 4 | Sunshine lag-1 (hours) | 1.80 × 10^-3 | 4.97 × 10^-4 | <0.001 |
| 4 | Sunshine lag-2 (hours) | 6.09 × 10^-4 | 4.83 × 10^-4 | 0.207 |
| 4 | Elevation (m) | 1.12 × 10^-3 | 9.48 × 10^-5 | <0.001 |
| 4 | NDVI | -7.23 × 10^-2 | 9.24 × 10^-2 | 0.434 |
| 4 | NDVI lag-1 | -2.56 × 10^-1 | 9.53 × 10^-2 | 0.007 |
| 4 | NDVI lag-2 | -3.00 × 10^-1 | 9.46 × 10^-2 | 0.002 |
| 4 | Standardised number of pigs per 100,000 population | 3.39 × 10^-1 | 1.47 × 10^-2 | <0.001 |
| 4 | Number of hospitals per 100 km^2^ | 3.01 × 10^-1 | 3.13 × 10^-2 | <0.001 |
| 4 | Poverty (%) | 3.53 × 10^-2 | 1.48 × 10^-3 | <0.001 |
| 4 | JEV vaccination coverage (%) | -1.61 × 10^-2 | 6.98 × 10^-4 | <0.001 |
| 5 | (Intercept) | -2.58 × 10^1 | 1.81 | <0.001 |
| 5 | sin(2 * pi * month2/12) | -1.59 × 10^-1 | 4.40 × 10^-2 | <0.001 |
| 5 | cos(2 * pi * month2/12) | 5.14 × 10^-3 | 3.95 × 10^-2 | 0.897 |
| 5 | Number of cases of meningitis per 100,000 population | 1.35 | 8.10 × 10^-2 | <0.001 |
| 5 | Number of cases of dengue fever per 100,000 population | -7.80 × 10^-4 | 1.08 × 10^-3 | 0.469 |
| 5 | Number of cases of ILI per 100,000 population | 2.27 × 10^-4 | 8.25 × 10^-5 | 0.006 |
| 5 | Number of cases of HFMD per 100,000 population | -1.19 × 10^-2 | 1.46 × 10^-3 | <0.001 |
| 5 | Number of cases of *S. suis* | 1.08 | 1.11 × 10^-1 | <0.001 |
| 5 | Proportion of children | 1.65 × 10^-1 | 6.43 × 10^-1 | 0.797 |
| 5 | Proportion of males | 1.67 × 10^1 | 3.43 | <0.001 |
| 5 | Minimum temperature (°C) | 6.65 × 10^-2 | 1.21 × 10^-2 | <0.001 |
| 5 | Minimum temperature lag-1 (°C) | 8.41 × 10^-2 | 1.49 × 10^-2 | <0.001 |
| 5 | Minimum temperature lag-2 (°C) | -9.40 × 10^-2 | 1.13 × 10^-2 | <0.001 |
| 5 | Relative humidity (%) | 2.09 × 10^-2 | 5.78 × 10^-3 | <0.001 |
| 5 | Relative humidity lag-1 (%) | 5.16 × 10^-3 | 6.39 × 10^-3 | 0.419 |
| 5 | Relative humidity lag-2 (%) | 3.07 × 10^-3 | 5.29 × 10^-3 | 0.562 |
| 5 | Rainfall (mm) | -5.77 × 10^-4 | 1.70 × 10^-4 | 0.001 |
| 5 | Rainfall lag-1 (mm) | 3.98 × 10^-4 | 1.77 × 10^-4 | 0.025 |
| 5 | Rainfall lag-2 (mm) | 8.04 × 10^-4 | 1.78 × 10^-4 | <0.001 |
| 5 | Rainfall lag-3 (mm) | -7.08 × 10^-4 | 1.81 × 10^-4 | <0.001 |
| 5 | Sunshine (hours) | 9.68 × 10^-4 | 4.16 × 10^-4 | 0.02 |
| 5 | Sunshine lag-1 (hours) | 2.44 × 10^-3 | 4.35 × 10^-4 | <0.001 |
| 5 | Sunshine lag-2 (hours) | 7.08 × 10^-4 | 4.23 × 10^-4 | 0.094 |
| 5 | Elevation (m) | 1.22 × 10^-3 | 1.09 × 10^-4 | <0.001 |
| 5 | NDVI | -3.28 × 10^-2 | 9.18 × 10^-2 | 0.721 |
| 5 | NDVI lag-1 | -2.01 × 10^-1 | 9.42 × 10^-2 | 0.033 |
| 5 | NDVI lag-2 | -2.72 × 10^-1 | 9.39 × 10^-2 | 0.004 |
| 5 | Standardised number of pigs per 100,000 population | 3.47 × 10^-1 | 1.46 × 10^-2 | <0.001 |
| 5 | Number of hospitals per 100 km^2^ | 3.13 × 10^-1 | 3.09 × 10^-2 | <0.001 |
| 5 | Poverty (%) | 3.62 × 10^-2 | 1.47 × 10^-3 | <0.001 |
| 5 | JEV vaccination coverage (%) | -1.64 × 10^-2 | 7.01 × 10^-4 | <0.001 |
| 6 | (Intercept) | -2.52 × 10^1 | 1.81 | <0.001 |
| 6 | sin(2 * pi * month2/12) | -2.78 × 10^-2 | 4.64 × 10^-2 | 0.549 |
| 6 | cos(2 * pi * month2/12) | 1.61 × 10^-2 | 3.99 × 10^-2 | 0.687 |
| 6 | Number of cases of meningitis per 100,000 population | 1.32 | 8.09 × 10^-2 | <0.001 |
| 6 | Number of cases of dengue fever per 100,000 population | -9.04 × 10^-4 | 1.07 × 10^-3 | 0.398 |
| 6 | Number of cases of ILI per 100,000 population | 2.37 × 10^-4 | 8.26 × 10^-5 | 0.004 |
| 6 | Number of cases of HFMD per 100,000 population | -1.07 × 10^-2 | 1.46 × 10^-3 | <0.001 |
| 6 | Number of cases of *S. suis* | 1.10 | 1.12 × 10^-1 | <0.001 |
| 6 | Proportion of children | 8.48 × 10^-1 | 6.50 × 10^-1 | 0.192 |
| 6 | Proportion of males | 1.61 × 10^1 | 3.44 | <0.001 |
| 6 | Absolute humidity (g/m3) | 6.40 × 10^-2 | 8.91 × 10^-3 | <0.001 |
| 6 | Absolute humidity lag-1 (g/m3) | 3.75 × 10^-2 | 1.10 × 10^-2 | 0.001 |
| 6 | Absolute humidity lag-2 (g/m3) | -3.14 × 10^-2 | 8.71 × 10^-3 | <0.001 |
| 6 | Relative humidity (%) | 8.15 × 10^-3 | 5.98 × 10^-3 | 0.173 |
| 6 | Relative humidity lag-1 (%) | 4.62 × 10^-3 | 6.75 × 10^-3 | 0.494 |
| 6 | Relative humidity lag-2 (%) | 5.63 × 10^-3 | 5.45 × 10^-3 | 0.302 |
| 6 | Rainfall (mm) | -5.01 × 10^-4 | 1.69 × 10^-4 | 0.003 |
| 6 | Rainfall lag-1 (mm) | 3.01 × 10^-4 | 1.76 × 10^-4 | 0.087 |
| 6 | Rainfall lag-2 (mm) | 7.86 × 10^-4 | 1.77 × 10^-4 | <0.001 |
| 6 | Rainfall lag-3 (mm) | -6.51 × 10^-4 | 1.78 × 10^-4 | <0.001 |
| 6 | Sunshine (hours) | 4.63 × 10^-4 | 4.17 × 10^-4 | 0.266 |
| 6 | Sunshine lag-1 (hours) | 2.26 × 10^-3 | 4.39 × 10^-4 | <0.001 |
| 6 | Sunshine lag-2 (hours) | 2.68 × 10^-4 | 4.36 × 10^-4 | 0.538 |
| 6 | Elevation (m) | 1.37 × 10^-3 | 9.97 × 10^-5 | <0.001 |
| 6 | NDVI | -7.91 × 10^-2 | 9.17 × 10^-2 | 0.388 |
| 6 | NDVI lag-1 | -2.21 × 10^-1 | 9.42 × 10^-2 | 0.019 |
| 6 | NDVI lag-2 | -2.93 × 10^-1 | 9.39 × 10^-2 | 0.002 |
| 6 | Standardised number of pigs per 100,000 population | 3.32 × 10^-1 | 1.47 × 10^-2 | <0.001 |
| 6 | Number of hospitals per 100 km^2^ | 3.14 × 10^-1 | 3.07 × 10^-2 | <0.001 |
| 6 | Poverty (%) | 3.72 × 10^-2 | 1.49 × 10^-3 | <0.001 |
| 6 | JEV vaccination coverage (%) | -1.68 × 10^-2 | 7.08 × 10^-4 | <0.001 |
| 7 | (Intercept) | -1.70 × 10^1 | 9.33 × 10^-1 | <0.001 |
| 7 | sin(2 * pi * month2/12) | 6.94 × 10^-2 | 2.34 × 10^-2 | 0.003 |
| 7 | cos(2 * pi * month2/12) | -2.38 × 10^-1 | 2.15 × 10^-2 | <0.001 |
| 7 | Proportion of children | 5.15 | 2.60 × 10^-1 | <0.001 |
| 7 | Proportion of males | -1.42 × 10^1 | 1.63 | <0.001 |
| 7 | Maximum temperature (°C) | 2.73 × 10^-2 | 6.63 × 10^-3 | <0.001 |
| 7 | Maximum temperature lag-1 (°C) | 8.08 × 10^-2 | 8.26 × 10^-3 | <0.001 |
| 7 | Maximum temperature lag-2 (°C) | 2.24 × 10^-3 | 6.49 × 10^-3 | 0.73 |
| 7 | Relative humidity (%) | 2.85 × 10^-2 | 2.82 × 10^-3 | <0.001 |
| 7 | Relative humidity lag-1 (%) | 1.95 × 10^-2 | 3.17 × 10^-3 | <0.001 |
| 7 | Relative humidity lag-2 (%) | 2.81 × 10^-2 | 2.75 × 10^-3 | <0.001 |
| 7 | Rainfall (mm) | -4.77 × 10^-4 | 9.04 × 10^-5 | <0.001 |
| 7 | Rainfall lag-1 (mm) | 6.91 × 10^-4 | 9.65 × 10^-5 | <0.001 |
| 7 | Rainfall lag-2 (mm) | -6.19 × 10^-4 | 1.02 × 10^-4 | <0.001 |
| 7 | Rainfall lag-3 (mm) | -6.27 × 10^-4 | 1.02 × 10^-4 | <0.001 |
| 7 | Sunshine (hours) | 1.03 × 10^-3 | 2.59 × 10^-4 | <0.001 |
| 7 | Sunshine lag-1 (hours) | 7.45 × 10^-4 | 2.62 × 10^-4 | 0.005 |
| 7 | Sunshine lag-2 (hours) | -2.05 × 10^-3 | 2.60 × 10^-4 | <0.001 |
| 7 | Elevation (m) | 7.27 × 10^-4 | 5.28 × 10^-5 | <0.001 |
| 7 | NDVI | -2.48 × 10^-1 | 5.43 × 10^-2 | <0.001 |
| 7 | NDVI lag-1 | 3.77 × 10^-1 | 5.27 × 10^-2 | <0.001 |
| 7 | NDVI lag-2 | 8.66 × 10^-3 | 5.49 × 10^-2 | 0.875 |
| 7 | Standardised number of pigs per 100,000 population | 2.72 × 10^-1 | 8.80 × 10^-3 | <0.001 |
| 7 | Number of hospitals per 100 km^2^ | 2.73 × 10^-2 | 1.91 × 10^-2 | 0.153 |
| 7 | Poverty (%) | 1.51 × 10^-2 | 6.83 × 10^-4 | <0.001 |
| 7 | JEV vaccination coverage (%) | -5.88 × 10^-3 | 3.67 × 10^-4 | <0.001 |
| 8 | (Intercept) | -1.58 × 10^1 | 9.20 × 10^-1 | <0.001 |
| 8 | sin(2 * pi * month2/12) | 5.75 × 10^-2 | 2.42 × 10^-2 | 0.017 |
| 8 | cos(2 * pi * month2/12) | -2.14 × 10^-1 | 2.23 × 10^-2 | <0.001 |
| 8 | Proportion of children | 4.93 | 2.59 × 10^-1 | <0.001 |
| 8 | Proportion of males | -1.43 × 10^1 | 1.63 | <0.001 |
| 8 | Minimum temperature (°C) | 3.81 × 10^-2 | 6.73 × 10^-3 | <0.001 |
| 8 | Minimum temperature lag-1 (°C) | 7.02 × 10^-2 | 8.19 × 10^-3 | <0.001 |
| 8 | Minimum temperature lag-2 (°C) | -3.63 × 10^-3 | 6.46 × 10^-3 | 0.574 |
| 8 | Relative humidity (%) | 2.62 × 10^-2 | 2.82 × 10^-3 | <0.001 |
| 8 | Relative humidity lag-1 (%) | 1.37 × 10^-2 | 3.15 × 10^-3 | <0.001 |
| 8 | Relative humidity lag-2 (%) | 2.93 × 10^-2 | 2.69 × 10^-3 | <0.001 |
| 8 | Rainfall (mm) | -4.34 × 10^-4 | 8.95 × 10^-5 | <0.001 |
| 8 | Rainfall lag-1 (mm) | 6.61 × 10^-4 | 9.57 × 10^-5 | <0.001 |
| 8 | Rainfall lag-2 (mm) | -6.58 × 10^-4 | 1.02 × 10^-4 | <0.001 |
| 8 | Rainfall lag-3 (mm) | -6.55 × 10^-4 | 1.02 × 10^-4 | <0.001 |
| 8 | Sunshine (hours) | 1.44 × 10^-3 | 2.23 × 10^-4 | <0.001 |
| 8 | Sunshine lag-1 (hours) | 1.81 × 10^-3 | 2.32 × 10^-4 | <0.001 |
| 8 | Sunshine lag-2 (hours) | -1.83 × 10^-3 | 2.26 × 10^-4 | <0.001 |
| 8 | Elevation (m) | 8.98 × 10^-4 | 6.11 × 10^-5 | <0.001 |
| 8 | NDVI | -2.22 × 10^-1 | 5.39 × 10^-2 | <0.001 |
| 8 | NDVI lag-1 | 4.49 × 10^-1 | 5.22 × 10^-2 | <0.001 |
| 8 | NDVI lag-2 | 5.91 × 10^-2 | 5.45 × 10^-2 | 0.278 |
| 8 | Standardised number of pigs per 100,000 population | 2.90 × 10^-1 | 8.78 × 10^-3 | <0.001 |
| 8 | Number of hospitals per 100 km^2^ | 4.82 × 10^-2 | 1.90 × 10^-2 | 0.011 |
| 8 | Poverty (%) | 1.64 × 10^-2 | 6.79 × 10^-4 | <0.001 |
| 8 | JEV vaccination coverage (%) | -6.15 × 10^-3 | 3.70 × 10^-4 | <0.001 |
| 9 | (Intercept) | -1.40 × 10^1 | 9.17 × 10^-1 | <0.001 |
| 9 | sin(2 * pi * month2/12) | 9.66 × 10^-2 | 2.60 × 10^-2 | <0.001 |
| 9 | cos(2 * pi * month2/12) | -1.68 × 10^-1 | 2.31 × 10^-2 | <0.001 |
| 9 | Proportion of children | 5.14 | 2.60 × 10^-1 | <0.001 |
| 9 | Proportion of males | -1.54 × 10^1 | 1.63 | <0.001 |
| 9 | Absolute humidity (g/m3) | 4.30 × 10^-2 | 5.08 × 10^-3 | <0.001 |
| 9 | Absolute humidity lag-1 (g/m3) | 4.19 × 10^-2 | 6.08 × 10^-3 | <0.001 |
| 9 | Absolute humidity lag-2 (g/m3) | 2.03 × 10^-3 | 4.99 × 10^-3 | 0.684 |
| 9 | Relative humidity (%) | 1.91 × 10^-2 | 2.99 × 10^-3 | <0.001 |
| 9 | Relative humidity lag-1 (%) | 8.49 × 10^-3 | 3.34 × 10^-3 | 0.011 |
| 9 | Relative humidity lag-2 (%) | 2.73 × 10^-2 | 2.77 × 10^-3 | <0.001 |
| 9 | Rainfall (mm) | -4.77 × 10^-4 | 9.04 × 10^-5 | <0.001 |
| 9 | Rainfall lag-1 (mm) | 5.80 × 10^-4 | 9.58 × 10^-5 | <0.001 |
| 9 | Rainfall lag-2 (mm) | -6.12 × 10^-4 | 1.03 × 10^-4 | <0.001 |
| 9 | Rainfall lag-3 (mm) | -5.71 × 10^-4 | 1.01 × 10^-4 | <0.001 |
| 9 | Sunshine (hours) | 1.20 × 10^-3 | 2.29 × 10^-4 | <0.001 |
| 9 | Sunshine lag-1 (hours) | 1.66 × 10^-3 | 2.35 × 10^-4 | <0.001 |
| 9 | Sunshine lag-2 (hours) | -1.72 × 10^-3 | 2.34 × 10^-4 | <0.001 |
| 9 | Elevation (m) | 9.44 × 10^-4 | 6.00 × 10^-5 | <0.001 |
| 9 | NDVI | -2.63 × 10^-1 | 5.42 × 10^-2 | <0.001 |
| 9 | NDVI lag-1 | 4.19 × 10^-1 | 5.23 × 10^-2 | <0.001 |
| 9 | NDVI lag-2 | 5.70 × 10^-2 | 5.45 × 10^-2 | 0.296 |
| 9 | Standardised number of pigs per 100,000 population | 2.78 × 10^-1 | 8.74 × 10^-3 | <0.001 |
| 9 | Number of hospitals per 100 km^2^ | 4.63 × 10^-2 | 1.91 × 10^-2 | 0.015 |
| 9 | Poverty (%) | 1.66 × 10^-2 | 6.81 × 10^-4 | <0.001 |
| 9 | JEV vaccination coverage (%) | -6.32 × 10^-3 | 3.72 × 10^-4 | <0.001 |

Table S5. Output of the ‘dispersiontest’ function for each of the Poisson linear mixed models.

| Model | Alpha | p value | Over-dispersed |
| --- | --- | --- | --- |
| 1 | 7.76 | <0.001 | Yes |
| 2 | 7.81 | <0.001 | Yes |
| 3 | 7.88 | <0.001 | Yes |
| 4 | 5.44 | <0.001 | Yes |
| 5 | 5.32 | <0.001 | Yes |
| 6 | 5.38 | <0.001 | Yes |
| 7 | 7.89 | <0.001 | Yes |
| 8 | 7.92 | <0.001 | Yes |
| 9 | 8.01 | <0.001 | Yes |

Table S6. Negative binomial linear mixed models showing the association between the number of cases of AES and each of the covariates.

| **Model** | **Covariate** | **Estimate** | **Standard error** | **p value** |
| --- | --- | --- | --- | --- |
| 1 | (Intercept) | 2.09 | 6.31 × 10^-1 | 0.001 |
| 1 | sin(2 * pi * month2/12) | 1.63 × 10^-1 | 1.54 × 10^-2 | <0.001 |
| 1 | cos(2 * pi * month2/12) | -1.32 × 10^-1 | 1.43 × 10^-2 | <0.001 |
| 1 | Number of cases of meningitis per 100,000 population | 1.22 | 2.94 × 10^-2 | <0.001 |
| 1 | Number of cases of dengue fever per 100,000 population | 2.47 × 10^-3 | 3.83 × 10^-4 | <0.001 |
| 1 | Number of cases of ILI per 100,000 population | 2.40 × 10^-4 | 3.81 × 10^-5 | <0.001 |
| 1 | Proportion of children | 1.48 | 1.91 × 10^-1 | <0.001 |
| 1 | Proportion of males | -2.24 × 10^1 | 1.12 | <0.001 |
| 1 | Maximum temperature (°C) | 6.04 × 10^-2 | 4.37 × 10^-3 | <0.001 |
| 1 | Maximum temperature lag-1 (°C) | 7.22 × 10^-2 | 5.66 × 10^-3 | <0.001 |
| 1 | Maximum temperature lag-2 (°C) | 9.38 × 10^-3 | 4.45 × 10^-3 | 0.035 |
| 1 | Relative humidity (%) | 3.17 × 10^-2 | 2.04 × 10^-3 | <0.001 |
| 1 | Relative humidity lag-1 (%) | 9.33 × 10^-3 | 2.25 × 10^-3 | <0.001 |
| 1 | Relative humidity lag-2 (%) | 2.57 × 10^-2 | 1.98 × 10^-3 | <0.001 |
| 1 | Rainfall (mm) | -3.59 × 10^-4 | 6.72 × 10^-5 | <0.001 |
| 1 | Rainfall lag-1 (mm) | 1.64 × 10^-4 | 7.14 × 10^-5 | 0.022 |
| 1 | Rainfall lag-2 (mm) | -3.42 × 10^-4 | 7.11 × 10^-5 | <0.001 |
| 1 | Rainfall lag-3 (mm) | -4.65 × 10^-4 | 6.47 × 10^-5 | <0.001 |
| 1 | Sunshine (hours) | 2.41 × 10^-4 | 1.94 × 10^-4 | 0.213 |
| 1 | Sunshine lag-1 (hours) | 1.70 × 10^-5 | 1.96 × 10^-4 | 0.931 |
| 1 | Sunshine lag-2 (hours) | -1.54 × 10^-3 | 1.92 × 10^-4 | <0.001 |
| 1 | Elevation (m) | 6.37 × 10^-4 | 3.92 × 10^-5 | <0.001 |
| 1 | NDVI | -3.29 × 10^-1 | 4.11 × 10^-2 | <0.001 |
| 1 | NDVI lag-1 | 2.78 × 10^-2 | 4.10 × 10^-2 | 0.498 |
| 1 | NDVI lag-2 | 8.44 × 10^-2 | 4.10 × 10^-2 | 0.039 |
| 1 | Standardised number of pigs per 100,000 population | 7.16 × 10^-2 | 6.93 × 10^-3 | <0.001 |
| 1 | Number of hospitals per 100 km^2^ | 3.47 × 10^-1 | 1.49 × 10^-2 | <0.001 |
| 1 | Poverty (%) | 2.57 × 10^-2 | 6.81 × 10^-4 | <0.001 |
| 1 | JEV vaccination coverage (%) | -8.51 × 10^-3 | 3.06 × 10^-4 | <0.001 |
| 2 | (Intercept) | 4.88 | 6.24 × 10^-1 | <0.001 |
| 2 | sin(2 * pi * month2/12) | 1.13 × 10^-1 | 1.60 × 10^-2 | <0.001 |
| 2 | cos(2 * pi * month2/12) | -1.75 × 10^-1 | 1.47 × 10^-2 | <0.001 |
| 2 | Number of cases of meningitis per 100,000 population | 1.20 | 2.94 × 10^-2 | <0.001 |
| 2 | Number of cases of dengue fever per 100,000 population | 2.67 × 10^-3 | 3.83 × 10^-4 | <0.001 |
| 2 | Number of cases of ILI per 100,000 population | 2.45 × 10^-4 | 3.82 × 10^-5 | <0.001 |
| 2 | Proportion of children | 9.55 × 10^-1 | 1.90 × 10^-1 | <0.001 |
| 2 | Proportion of males | -2.44 × 10^1 | 1.13 | <0.001 |
| 2 | Minimum temperature (°C) | 3.78 × 10^-2 | 4.38 × 10^-3 | <0.001 |
| 2 | Minimum temperature lag-1 (°C) | 7.49 × 10^-2 | 5.44 × 10^-3 | <0.001 |
| 2 | Minimum temperature lag-2 (°C) | -9.44 × 10^-3 | 4.43 × 10^-3 | 0.033 |
| 2 | Relative humidity (%) | 2.83 × 10^-2 | 2.07 × 10^-3 | <0.001 |
| 2 | Relative humidity lag-1 (%) | 3.50 × 10^-3 | 2.29 × 10^-3 | 0.127 |
| 2 | Relative humidity lag-2 (%) | 3.11 × 10^-2 | 1.97 × 10^-3 | <0.001 |
| 2 | Rainfall (mm) | -2.73 × 10^-4 | 6.69 × 10^-5 | <0.001 |
| 2 | Rainfall lag-1 (mm) | 2.13 × 10^-4 | 7.14 × 10^-5 | 0.003 |
| 2 | Rainfall lag-2 (mm) | -3.78 × 10^-4 | 7.16 × 10^-5 | <0.001 |
| 2 | Rainfall lag-3 (mm) | -4.88 × 10^-4 | 6.52 × 10^-5 | <0.001 |
| 2 | Sunshine (hours) | 1.63 × 10^-3 | 1.71 × 10^-4 | <0.001 |
| 2 | Sunshine lag-1 (hours) | 1.33 × 10^-3 | 1.76 × 10^-4 | <0.001 |
| 2 | Sunshine lag-2 (hours) | -1.16 × 10^-3 | 1.70 × 10^-4 | <0.001 |
| 2 | Elevation (m) | 6.85 × 10^-4 | 4.55 × 10^-5 | <0.001 |
| 2 | NDVI | -2.38 × 10^-1 | 4.07 × 10^-2 | <0.001 |
| 2 | NDVI lag-1 | 1.64 × 10^-1 | 4.07 × 10^-2 | <0.001 |
| 2 | NDVI lag-2 | 2.13 × 10^-1 | 4.06 × 10^-2 | <0.001 |
| 2 | Standardised number of pigs per 100,000 population | 8.72 × 10^-2 | 6.93 × 10^-3 | <0.001 |
| 2 | Number of hospitals per 100 km^2^ | 3.44 × 10^-1 | 1.49 × 10^-2 | <0.001 |
| 2 | Poverty (%) | 2.71 × 10^-2 | 6.81 × 10^-4 | <0.001 |
| 2 | JEV vaccination coverage (%) | -8.97 × 10^-3 | 3.08 × 10^-4 | <0.001 |
| 3 | (Intercept) | 6.43 | 6.23 × 10^-1 | <0.001 |
| 3 | sin(2 * pi * month2/12) | 1.24 × 10^-1 | 1.72 × 10^-2 | <0.001 |
| 3 | cos(2 * pi * month2/12) | -1.63 × 10^-1 | 1.54 × 10^-2 | <0.001 |
| 3 | Number of cases of meningitis per 100,000 population | 1.21 | 2.95 × 10^-2 | <0.001 |
| 3 | Number of cases of dengue fever per 100,000 population | 2.41 × 10^-3 | 3.83 × 10^-4 | <0.001 |
| 3 | Number of cases of ILI per 100,000 population | 2.65 × 10^-4 | 3.83 × 10^-5 | <0.001 |
| 3 | Proportion of children | 1.06 | 1.92 × 10^-1 | <0.001 |
| 3 | Proportion of males | -2.53 × 10^1 | 1.13 | <0.001 |
| 3 | Absolute humidity (g/m3) | 3.06 × 10^-2 | 3.39 × 10^-3 | <0.001 |
| 3 | Absolute humidity lag-1 (g/m3) | 4.66 × 10^-2 | 4.16 × 10^-3 | <0.001 |
| 3 | Absolute humidity lag-2 (g/m3) | -5.28 × 10^-3 | 3.46 × 10^-3 | 0.128 |
| 3 | Relative humidity (%) | 2.46 × 10^-2 | 2.19 × 10^-3 | <0.001 |
| 3 | Relative humidity lag-1 (%) | -1.19 × 10^-3 | 2.45 × 10^-3 | 0.627 |
| 3 | Relative humidity lag-2 (%) | 2.99 × 10^-2 | 2.06 × 10^-3 | <0.001 |
| 3 | Rainfall (mm) | -2.76 × 10^-4 | 6.75 × 10^-5 | <0.001 |
| 3 | Rainfall lag-1 (mm) | 1.63 × 10^-4 | 7.15 × 10^-5 | 0.023 |
| 3 | Rainfall lag-2 (mm) | -3.06 × 10^-4 | 7.19 × 10^-5 | <0.001 |
| 3 | Rainfall lag-3 (mm) | -3.95 × 10^-4 | 6.49 × 10^-5 | <0.001 |
| 3 | Sunshine (hours) | 1.82 × 10^-3 | 1.74 × 10^-4 | <0.001 |
| 3 | Sunshine lag-1 (hours) | 1.40 × 10^-3 | 1.78 × 10^-4 | <0.001 |
| 3 | Sunshine lag-2 (hours) | -1.06 × 10^-3 | 1.75 × 10^-4 | <0.001 |
| 3 | Elevation (m) | 5.99 × 10^-4 | 4.47 × 10^-5 | <0.001 |
| 3 | NDVI | -2.60 × 10^-1 | 4.09 × 10^-2 | <0.001 |
| 3 | NDVI lag-1 | 1.45 × 10^-1 | 4.08 × 10^-2 | <0.001 |
| 3 | NDVI lag-2 | 2.22 × 10^-1 | 4.08 × 10^-2 | <0.001 |
| 3 | Standardised number of pigs per 100,000 population | 7.92 × 10^-2 | 6.92 × 10^-3 | <0.001 |
| 3 | Number of hospitals per 100 km^2^ | 3.53 × 10^-1 | 1.49 × 10^-2 | <0.001 |
| 3 | Poverty (%) | 2.70 × 10^-2 | 6.82 × 10^-4 | <0.001 |
| 3 | JEV vaccination coverage (%) | -9.15 × 10^-3 | 3.09 × 10^-4 | <0.001 |
| 4 | (Intercept) | -8.82 | 1.14 | <0.001 |
| 4 | sin(2 * pi * month2/12) | 1.41 × 10^-1 | 2.63 × 10^-2 | <0.001 |
| 4 | cos(2 * pi * month2/12) | -6.92 × 10^-2 | 2.41 × 10^-2 | 0.004 |
| 4 | Number of cases of meningitis per 100,000 population | 3.27 | 1.30 × 10^-1 | <0.001 |
| 4 | Number of cases of dengue fever per 100,000 population | 3.54 × 10^-3 | 6.68 × 10^-4 | <0.001 |
| 4 | Number of cases of ILI per 100,000 population | -4.29 × 10^-4 | 8.20 × 10^-5 | <0.001 |
| 4 | Number of cases of HFMD per 100,000 population | -8.73 × 10^-3 | 8.17 × 10^-4 | <0.001 |
| 4 | Number of cases of *S. suis* | 2.20 | 1.75 × 10^-1 | <0.001 |
| 4 | Proportion of children | -1.07 | 4.41 × 10^-1 | 0.015 |
| 4 | Proportion of males | 2.87 | 2.14 | 0.181 |
| 4 | Maximum temperature (°C) | 8.47 × 10^-2 | 7.60 × 10^-3 | <0.001 |
| 4 | Maximum temperature lag-1 (°C) | 7.81 × 10^-2 | 9.90 × 10^-3 | <0.001 |
| 4 | Maximum temperature lag-2 (°C) | 3.29 × 10^-3 | 7.67 × 10^-3 | 0.668 |
| 4 | Relative humidity (%) | 3.42 × 10^-2 | 3.74 × 10^-3 | <0.001 |
| 4 | Relative humidity lag-1 (%) | 5.53 × 10^-4 | 4.00 × 10^-3 | 0.89 |
| 4 | Relative humidity lag-2 (%) | 1.16 × 10^-2 | 3.60 × 10^-3 | 0.001 |
| 4 | Rainfall (mm) | -4.35 × 10^-4 | 1.17 × 10^-4 | <0.001 |
| 4 | Rainfall lag-1 (mm) | 3.08 × 10^-4 | 1.22 × 10^-4 | 0.011 |
| 4 | Rainfall lag-2 (mm) | 1.06 × 10^-4 | 1.21 × 10^-4 | 0.382 |
| 4 | Rainfall lag-3 (mm) | 2.28 × 10^-5 | 1.09 × 10^-4 | 0.834 |
| 4 | Sunshine (hours) | -9.11 × 10^-4 | 3.44 × 10^-4 | 0.008 |
| 4 | Sunshine lag-1 (hours) | -1.72 × 10^-4 | 3.52 × 10^-4 | 0.625 |
| 4 | Sunshine lag-2 (hours) | -3.27 × 10^-4 | 3.39 × 10^-4 | 0.335 |
| 4 | Elevation (m) | 1.10 × 10^-3 | 6.95 × 10^-5 | <0.001 |
| 4 | NDVI | -3.75 × 10^-1 | 6.76 × 10^-2 | <0.001 |
| 4 | NDVI lag-1 | -7.13 × 10^-1 | 6.91 × 10^-2 | <0.001 |
| 4 | NDVI lag-2 | -4.34 × 10^-1 | 6.79 × 10^-2 | <0.001 |
| 4 | Standardised number of pigs per 100,000 population | 1.40 × 10^-1 | 1.07 × 10^-2 | <0.001 |
| 4 | Number of hospitals per 100 km^2^ | 6.83 × 10^-1 | 2.43 × 10^-2 | <0.001 |
| 4 | Poverty (%) | 5.28 × 10^-2 | 1.37 × 10^-3 | <0.001 |
| 4 | JEV vaccination coverage (%) | -1.48 × 10^-2 | 5.26 × 10^-4 | <0.001 |
| 5 | (Intercept) | -6.53 | 1.13 | <0.001 |
| 5 | sin(2 * pi * month2/12) | -1.31 × 10^-2 | 2.74 × 10^-2 | 0.631 |
| 5 | cos(2 * pi * month2/12) | -9.81 × 10^-2 | 2.46 × 10^-2 | <0.001 |
| 5 | Number of cases of meningitis per 100,000 population | 3.17 | 1.31 × 10^-1 | <0.001 |
| 5 | Number of cases of dengue fever per 100,000 population | 3.61 × 10^-3 | 6.66 × 10^-4 | <0.001 |
| 5 | Number of cases of ILI per 100,000 population | -4.16 × 10^-4 | 8.23 × 10^-5 | <0.001 |
| 5 | Number of cases of HFMD per 100,000 population | -1.02 × 10^-2 | 8.19 × 10^-4 | <0.001 |
| 5 | Number of cases of *S. suis* | 2.27 | 1.76 × 10^-1 | <0.001 |
| 5 | Proportion of children | -2.19 | 4.34 × 10^-1 | <0.001 |
| 5 | Proportion of males | 3.89 | 2.14 | 0.069 |
| 5 | Minimum temperature (°C) | 6.04 × 10^-2 | 7.77 × 10^-3 | <0.001 |
| 5 | Minimum temperature lag-1 (°C) | 9.42 × 10^-2 | 9.86 × 10^-3 | <0.001 |
| 5 | Minimum temperature lag-2 (°C) | -6.73 × 10^-2 | 7.74 × 10^-3 | <0.001 |
| 5 | Relative humidity (%) | 2.82 × 10^-2 | 3.81 × 10^-3 | <0.001 |
| 5 | Relative humidity lag-1 (%) | -9.23 × 10^-3 | 4.13 × 10^-3 | 0.025 |
| 5 | Relative humidity lag-2 (%) | 2.16 × 10^-2 | 3.58 × 10^-3 | <0.001 |
| 5 | Rainfall (mm) | -3.82 × 10^-4 | 1.16 × 10^-4 | 0.001 |
| 5 | Rainfall lag-1 (mm) | 4.54 × 10^-4 | 1.22 × 10^-4 | <0.001 |
| 5 | Rainfall lag-2 (mm) | 1.39 × 10^-4 | 1.21 × 10^-4 | 0.253 |
| 5 | Rainfall lag-3 (mm) | 1.21 × 10^-4 | 1.10 × 10^-4 | 0.272 |
| 5 | Sunshine (hours) | 1.02 × 10^-3 | 2.99 × 10^-4 | 0.001 |
| 5 | Sunshine lag-1 (hours) | 1.36 × 10^-3 | 3.08 × 10^-4 | <0.001 |
| 5 | Sunshine lag-2 (hours) | 9.52 × 10^-4 | 2.99 × 10^-4 | 0.001 |
| 5 | Elevation (m) | 9.73 × 10^-4 | 8.00 × 10^-5 | <0.001 |
| 5 | NDVI | -2.37 × 10^-1 | 6.71 × 10^-2 | <0.001 |
| 5 | NDVI lag-1 | -5.58 × 10^-1 | 6.82 × 10^-2 | <0.001 |
| 5 | NDVI lag-2 | -2.43 × 10^-1 | 6.71 × 10^-2 | <0.001 |
| 5 | Standardised number of pigs per 100,000 population | 1.57 × 10^-1 | 1.06 × 10^-2 | <0.001 |
| 5 | Number of hospitals per 100 km22 | 7.15 × 10^-1 | 2.42 × 10^-2 | <0.001 |
| 5 | Poverty (%) | 5.45 × 10^-2 | 1.37 × 10^-3 | <0.001 |
| 5 | JEV vaccination coverage (%) | -1.53 × 10^-2 | 5.27 × 10^-4 | <0.001 |
| 6 | (Intercept) | -6.14 | 1.13 | <0.001 |
| 6 | sin(2 * pi * month2/12) | 3.29 × 10^-2 | 2.90 × 10^-2 | 0.258 |
| 6 | cos(2 * pi * month2/12) | -8.75 × 10^-2 | 2.55 × 10^-2 | 0.001 |
| 6 | Number of cases of meningitis per 100,000 population | 3.27 | 1.31 × 10^-1 | <0.001 |
| 6 | Number of cases of dengue fever per 100,000 population | 3.64 × 10^-3 | 6.65 × 10^-4 | <0.001 |
| 6 | Number of cases of ILI per 100,000 population | -3.41 × 10^-4 | 8.15 × 10^-5 | <0.001 |
| 6 | Number of cases of HFMD per 100,000 population | -1.01 × 10^-2 | 8.23 × 10^-4 | <0.001 |
| 6 | Number of cases of *S. suis* | 2.17 | 1.76 × 10^-1 | <0.001 |
| 6 | Proportion of children | -1.92 | 4.38 × 10^-1 | <0.001 |
| 6 | Proportion of males | 4.55 | 2.14 | 0.034 |
| 6 | Absolute humidity (g/m3) | 4.70 × 10^-2 | 5.92 × 10^-3 | <0.001 |
| 6 | Absolute humidity lag-1 (g/m3) | 6.13 × 10^-2 | 7.46 × 10^-3 | <0.001 |
| 6 | Absolute humidity lag-2 (g/m3) | -3.90 × 10^-2 | 5.99 × 10^-3 | <0.001 |
| 6 | Relative humidity (%) | 2.21 × 10^-2 | 4.06 × 10^-3 | <0.001 |
| 6 | Relative humidity lag-1 (%) | -1.50 × 10^-2 | 4.47 × 10^-3 | 0.001 |
| 6 | Relative humidity lag-2 (%) | 2.51 × 10^-2 | 3.74 × 10^-3 | <0.001 |
| 6 | Rainfall (mm) | -3.50 × 10^-4 | 1.16 × 10^-4 | 0.003 |
| 6 | Rainfall lag-1 (mm) | 3.88 × 10^-4 | 1.22 × 10^-4 | 0.001 |
| 6 | Rainfall lag-2 (mm) | 1.99 × 10^-4 | 1.22 × 10^-4 | 0.102 |
| 6 | Rainfall lag-3 (mm) | 2.21 × 10^-4 | 1.10 × 10^-4 | 0.043 |
| 6 | Sunshine (hours) | 1.04 × 10^-3 | 3.02 × 10^-4 | 0.001 |
| 6 | Sunshine lag-1 (hours) | 1.38 × 10^-3 | 3.14 × 10^-4 | <0.001 |
| 6 | Sunshine lag-2 (hours) | 9.06 × 10^-4 | 3.11 × 10^-4 | 0.004 |
| 6 | Elevation (m) | 9.22 × 10^-4 | 7.52 × 10^-5 | <0.001 |
| 6 | NDVI | -2.82 × 10^-1 | 6.71 × 10^-2 | <0.001 |
| 6 | NDVI lag-1 | -5.49 × 10^-1 | 6.82 × 10^-2 | <0.001 |
| 6 | NDVI lag-2 | -2.61 × 10^-1 | 6.73 × 10^-2 | <0.001 |
| 6 | Standardised number of pigs per 100,000 population | 1.47 × 10^-1 | 1.07 × 10^-2 | <0.001 |
| 6 | Number of hospitals per 100 km^2^ | 7.43 × 10^-1 | 2.41 × 10^-2 | <0.001 |
| 6 | Poverty (%) | 5.48 × 10^-2 | 1.37 × 10^-3 | <0.001 |
| 6 | JEV vaccination coverage (%) | -1.58 × 10^-2 | 5.29 × 10^-4 | <0.001 |
| 7 | (Intercept) | 2.91 | 6.32 × 10^-1 | <0.001 |
| 7 | sin(2 * pi * month2/12) | 1.20 × 10^-1 | 1.54 × 10^-2 | <0.001 |
| 7 | cos(2 * pi * month2/12) | -1.38 × 10^-1 | 1.43 × 10^-2 | <0.001 |
| 7 | Proportion of children | 2.01 | 1.91 × 10^-1 | <0.001 |
| 7 | Proportion of males | -2.51 × 10^1 | 1.13 | <0.001 |
| 7 | Maximum temperature (°C) | 6.14 × 10^-2 | 4.39 × 10^-3 | <0.001 |
| 7 | Maximum temperature lag-1 (°C) | 7.94 × 10^-2 | 5.67 × 10^-3 | <0.001 |
| 7 | Maximum temperature lag-2 (°C) | 4.17 × 10^-3 | 4.47 × 10^-3 | 0.351 |
| 7 | Relative humidity (%) | 3.45 × 10^-2 | 2.04 × 10^-3 | <0.001 |
| 7 | Relative humidity lag-1 (%) | 1.24 × 10^-2 | 2.26 × 10^-3 | <0.001 |
| 7 | Relative humidity lag-2 (%) | 2.65 × 10^-2 | 1.99 × 10^-3 | <0.001 |
| 7 | Rainfall (mm) | -5.66 × 10^-4 | 6.75 × 10^-5 | <0.001 |
| 7 | Rainfall lag-1 (mm) | 1.74 × 10^-4 | 7.18 × 10^-5 | 0.016 |
| 7 | Rainfall lag-2 (mm) | -3.76 × 10^-4 | 7.14 × 10^-5 | <0.001 |
| 7 | Rainfall lag-3 (mm) | -5.49 × 10^-4 | 6.49 × 10^-5 | <0.001 |
| 7 | Sunshine (hours) | 2.48 × 10^-4 | 1.95 × 10^-4 | 0.203 |
| 7 | Sunshine lag-1 (hours) | -1.49 × 10^-4 | 1.96 × 10^-4 | 0.447 |
| 7 | Sunshine lag-2 (hours) | -1.27 × 10^-3 | 1.92 × 10^-4 | <0.001 |
| 7 | Elevation (m) | 7.72 × 10^-4 | 3.91 × 10^-5 | <0.001 |
| 7 | NDVI | -3.94 × 10^-1 | 4.12 × 10^-2 | <0.001 |
| 7 | NDVI lag-1 | 9.30 × 10^-3 | 4.11 × 10^-2 | 0.821 |
| 7 | NDVI lag-2 | 3.75 × 10^-2 | 4.11 × 10^-2 | 0.363 |
| 7 | Standardised number of pigs per 100,000 population | 6.98 × 10^-2 | 6.78 × 10^-3 | <0.001 |
| 7 | Number of hospitals per 100 km^2^ | 3.39 × 10^-1 | 1.49 × 10^-2 | <0.001 |
| 7 | Poverty (%) | 2.64 × 10^-2 | 6.34 × 10^-4 | <0.001 |
| 7 | JEV vaccination coverage (%) | -9.21 × 10^-3 | 3.04 × 10^-4 | <0.001 |
| 8 | (Intercept) | 5.72 | 6.25 × 10^-1 | <0.001 |
| 8 | sin(2 * pi * month2/12) | 7.14 × 10^-2 | 1.60 × 10^-2 | <0.001 |
| 8 | cos(2 * pi * month2/12) | -1.76 × 10^-1 | 1.47 × 10^-2 | <0.001 |
| 8 | Proportion of children | 1.48 | 1.90 × 10^-1 | <0.001 |
| 8 | Proportion of males | -2.72 × 10^1 | 1.13 | <0.001 |
| 8 | Minimum temperature (°C) | 4.03 × 10^-2 | 4.39 × 10^-3 | <0.001 |
| 8 | Minimum temperature lag-1 (°C) | 8.20 × 10^-2 | 5.45 × 10^-3 | <0.001 |
| 8 | Minimum temperature lag-2 (°C) | -1.41 × 10^-2 | 4.45 × 10^-3 | 0.002 |
| 8 | Relative humidity (%) | 3.08 × 10^-2 | 2.08 × 10^-3 | <0.001 |
| 8 | Relative humidity lag-1 (%) | 5.86 × 10^-3 | 2.30 × 10^-3 | 0.011 |
| 8 | Relative humidity lag-2 (%) | 3.25 × 10^-2 | 1.98 × 10^-3 | <0.001 |
| 8 | Rainfall (mm) | -4.86 × 10^-4 | 6.72 × 10^-5 | <0.001 |
| 8 | Rainfall lag-1 (mm) | 2.16 × 10^-4 | 7.18 × 10^-5 | 0.003 |
| 8 | Rainfall lag-2 (mm) | -4.22 × 10^-4 | 7.19 × 10^-5 | <0.001 |
| 8 | Rainfall lag-3 (mm) | -5.81 × 10^-4 | 6.55 × 10^-5 | <0.001 |
| 8 | Sunshine (hours) | 1.62 × 10^-3 | 1.71 × 10^-4 | <0.001 |
| 8 | Sunshine lag-1 (hours) | 1.21 × 10^-3 | 1.76 × 10^-4 | <0.001 |
| 8 | Sunshine lag-2 (hours) | -9.47 × 10^-4 | 1.70 × 10^-4 | <0.001 |
| 8 | Elevation (m) | 8.37 × 10^-4 | 4.54 × 10^-5 | <0.001 |
| 8 | NDVI | -3.02 × 10^-1 | 4.09 × 10^-2 | <0.001 |
| 8 | NDVI lag-1 | 1.50 × 10^-1 | 4.07 × 10^-2 | <0.001 |
| 8 | NDVI lag-2 | 1.70 × 10^-1 | 4.08 × 10^-2 | <0.001 |
| 8 | Standardised number of pigs per 100,000 population | 8.48 × 10^-2 | 6.78 × 10^-3 | <0.001 |
| 8 | Number of hospitals per 100 km^2^ | 3.32 × 10^-1 | 1.49 × 10^-2 | <0.001 |
| 8 | Poverty (%) | 2.78 × 10^-2 | 6.34 × 10^-4 | <0.001 |
| 8 | JEV vaccination coverage (%) | -9.69 × 10^-3 | 3.06 × 10^-4 | <0.001 |
| 9 | (Intercept) | 7.42 | 6.24 × 10^-1 | <0.001 |
| 9 | sin(2 * pi * month2/12) | 8.71 × 10^-2 | 1.72 × 10^-2 | <0.001 |
| 9 | cos(2 * pi * month2/12) | -1.65 × 10^-1 | 1.55 × 10^-2 | <0.001 |
| 9 | Proportion of children | 1.60 | 1.91 × 10^-1 | <0.001 |
| 9 | Proportion of males | -2.83 × 10^1 | 1.13 | <0.001 |
| 9 | Absolute humidity (g/m3) | 3.19 × 10^-2 | 3.40 × 10^-3 | <0.001 |
| 9 | Absolute humidity lag-1 (g/m3) | 5.17 × 10^-2 | 4.18 × 10^-3 | <0.001 |
| 9 | Absolute humidity lag-2 (g/m3) | -7.20 × 10^-3 | 3.48 × 10^-3 | 0.039 |
| 9 | Relative humidity (%) | 2.70 × 10^-2 | 2.20 × 10^-3 | <0.001 |
| 9 | Relative humidity lag-1 (%) | 6.43 × 10^-4 | 2.46 × 10^-3 | 0.794 |
| 9 | Relative humidity lag-2 (%) | 3.13 × 10^-2 | 2.07 × 10^-3 | <0.001 |
| 9 | Rainfall (mm) | -4.95 × 10^-4 | 6.78 × 10^-5 | <0.001 |
| 9 | Rainfall lag-1 (mm) | 1.53 × 10^-4 | 7.19 × 10^-5 | 0.033 |
| 9 | Rainfall lag-2 (mm) | -3.46 × 10^-4 | 7.22 × 10^-5 | <0.001 |
| 9 | Rainfall lag-3 (mm) | -4.86 × 10^-4 | 6.51 × 10^-5 | <0.001 |
| 9 | Sunshine (hours) | 1.81 × 10^-3 | 1.74 × 10^-4 | <0.001 |
| 9 | Sunshine lag-1 (hours) | 1.25 × 10^-3 | 1.78 × 10^-4 | <0.001 |
| 9 | Sunshine lag-2 (hours) | -8.88 × 10^-4 | 1.75 × 10^-4 | <0.001 |
| 9 | Elevation (m) | 7.66 × 10^-4 | 4.46 × 10^-5 | <0.001 |
| 9 | NDVI | -3.22 × 10^-1 | 4.10 × 10^-2 | <0.001 |
| 9 | NDVI lag-1 | 1.32 × 10^-1 | 4.08 × 10^-2 | 0.001 |
| 9 | NDVI lag-2 | 1.79 × 10^-1 | 4.09 × 10^-2 | <0.001 |
| 9 | Standardised number of pigs per 100,000 population | 7.72 × 10^-2 | 6.77 × 10^-3 | <0.001 |
| 9 | Number of hospitals per 100 km^2^ | 3.41 × 10^-1 | 1.49 × 10^-2 | <0.001 |
| 9 | Poverty (%) | 2.79 × 10^-2 | 6.34 × 10^-4 | <0.001 |
| 9 | JEV vaccination coverage (%) | -9.91 × 10^-3 | 3.07 × 10^-4 | <0.001 |

Table S7. Spatial autocorrelation amongst the residuals from the six negative binomial linear mixed models.

| **Model** | **Moran’s I** | **p value** | **Spatial autocorrelation of the residuals** |
| --- | --- | --- | --- |
| 1 | 6.31x10^-2^ | <0.001 | Yes |
| 2 | 6.49x10^-2^ | <0.001 | Yes |
| 3 | 6.50x10^-2^ | <0.001 | Yes |
| 4 | 5.39x10^-2^ | <0.001 | Yes |
| 5 | 5.74x10^-2^ | <0.001 | Yes |
| 6 | 5.72x10^-2^ | <0.001 | Yes |
| 7 | 6.78x10^-2^ | <0.001 | Yes |
| 8 | 6.96x10^-2^ | <0.001 | Yes |
| 9 | 6.97x10^-2^ | <0.001 | Yes |

Table S8. Temporal autocorrelation amongst the residuals from the six negative binomial linear mixed models.

| **Model** | **x-squared** | **p value** | **Temporal autocorrelation of the residuals** |
| --- | --- | --- | --- |
| 1 | 4.36 | 0.037 | Yes |
| 2 | 3.55 | 0.060 | No |
| 3 | 3.56 | 0.059 | No |
| 4 | 6.06 | 0.014 | Yes |
| 5 | 6.55 | 0.011 | Yes |
| 6 | 7.70 | 0.006 | Yes |
| 7 | 5.17 | 0.023 | Yes |
| 8 | 3.99 | 0.046 | Yes |
| 9 | 3.95 | 0.047 | Yes |

Table S9. The Watanabe-Akaike criterion (WAIC) and deviance information criterion (DIC) from the final spatio-temporal negative binomial models.

| **Model number** | **WAIC** | **DIC** |
| --- | --- | --- |
| 1 | 44239.37 | 43509.41 |
| 2 | 44246.07 | 43511.07 |
| 3 | 44271.88 | 43527.94 |
| 4 | 44224.01 | 43486.9 |
| 5 | 44224.64 | 43486.14 |
| 6 | 44261.77 | 43506.15 |
| 7 | 44552.26 | 43654.6 |
| 8 | 44491.43 | 43669.56 |
| 9 | 44552.8 | 43686.2 |

Table S10. Final spatio-temporal negative binomial models showing the association between the number of cases of AES and each of the covariates.

| **Model** | **Covariate** | **Mean posterior estimate** | **2.5% credible interval** | **97.5% credible interval** |
| --- | --- | --- | --- | --- |
| 1 | (Intercept) | -1.98 × 10^1 | -2.75 × 10^1 | -1.20 × 10^1 |
| 1 | sin(2 * pi * month2/12) | 3.51 × 10^-2 | -2.57 × 10^-1 | 3.26 × 10^-1 |
| 1 | cos(2 * pi * month2/12) | -1.39 × 10^-1 | -4.07 × 10^-1 | 1.33 × 10^-1 |
| 1 | Number of cases of meningitis per 100,000 population | 7.87 × 10^-1 | 6.14 × 10^-1 | 9.60 × 10^-1 |
| 1 | Number of cases of dengue fever per 100,000 population | 1.49 × 10^-3 | -1.28 × 10^-4 | 3.10 × 10^-3 |
| 1 | Number of cases of ILI per 100,000 population | 4.93 × 10^-4 | 2.51 × 10^-4 | 7.34 × 10^-4 |
| 1 | Proportion of children | 4.11 | 3.66 × 10^-1 | 7.84 |
| 1 | Proportion of males | 3.09 × 10^-1 | -1.51 × 10^1 | 1.57 × 10^1 |
| 1 | Maximum temperature (°C) | 6.65 × 10^-2 | 4.02 × 10^-2 | 9.28 × 10^-2 |
| 1 | Maximum temperature lag-1 (°C) | 6.02 × 10^-2 | 3.10 × 10^-2 | 8.95 × 10^-2 |
| 1 | Maximum temperature lag-2 (°C) | -3.10 × 10^-2 | -5.44 × 10^-2 | -7.55 × 10^-3 |
| 1 | Relative humidity (%) | 2.73 × 10^-2 | 1.47 × 10^-2 | 3.98 × 10^-2 |
| 1 | Relative humidity lag-1 (%) | -7.85 × 10^-3 | -1.65 × 10^-2 | 8.25 × 10^-4 |
| 1 | Relative humidity lag-2 (%) | 6.50 × 10^-3 | -2.73 × 10^-4 | 1.33 × 10^-2 |
| 1 | Rainfall (mm) | 1.41 × 10^-4 | -2.42 × 10^-4 | 5.24 × 10^-4 |
| 1 | Rainfall lag-1 (mm) | 3.41 × 10^-4 | -6.24 × 10^-5 | 7.45 × 10^-4 |
| 1 | Rainfall lag-2 (mm) | -5.64 × 10^-5 | -4.48 × 10^-4 | 3.35 × 10^-4 |
| 1 | Rainfall lag-3 (mm) | -4.94 × 10^-5 | -4.21 × 10^-4 | 3.22 × 10^-4 |
| 1 | Sunshine (hours) | 7.67 × 10^-4 | -3.92 × 10^-4 | 1.93 × 10^-3 |
| 1 | Sunshine lag-1 (hours) | -7.55 × 10^-4 | -1.95 × 10^-3 | 4.38 × 10^-4 |
| 1 | Sunshine lag-2 (hours) | -1.85 × 10^-3 | -3.03 × 10^-3 | -6.68 × 10^-4 |
| 1 | Elevation (m) | 8.68 × 10^-4 | -2.19 × 10^-4 | 1.96 × 10^-3 |
| 1 | NDVI | -5.89 × 10^-2 | -2.97 × 10^-1 | 1.79 × 10^-1 |
| 1 | NDVI lag-1 | 3.50 × 10^-1 | 1.14 × 10^-1 | 5.86 × 10^-1 |
| 1 | NDVI lag-2 | 1.47 × 10^-1 | -9.12 × 10^-2 | 3.85 × 10^-1 |
| 1 | Number of hospitals per 100 km22 | -1.30 × 10^-1 | -4.93 × 10^-1 | 2.32 × 10^-1 |
| 1 | Poverty (%) | -2.48 × 10^-2 | -3.53 × 10^-2 | -1.43 × 10^-2 |
| 1 | JEV vaccination coverage (%) | 2.37 × 10^-3 | 1.26 × 10^-3 | 3.48 × 10^-3 |
| 1 | Standardised number of pigs per 100,000 population | 1.68 × 10^-1 | 7.14 × 10^-2 | 2.65 × 10^-1 |
| 2 | (Intercept) | -1.88 × 10^1 | -2.66 × 10^1 | -1.10 × 10^1 |
| 2 | sin(2 * pi * month2/12) | 2.77 × 10^-2 | -2.59 × 10^-1 | 3.12 × 10^-1 |
| 2 | cos(2 * pi * month2/12) | -1.29 × 10^-1 | -3.90 × 10^-1 | 1.37 × 10^-1 |
| 2 | Number of cases of meningitis per 100,000 population | 7.87 × 10^-1 | 6.13 × 10^-1 | 9.60 × 10^-1 |
| 2 | Number of cases of dengue fever per 100,000 population | 1.54 × 10^-3 | -8.10 × 10^-5 | 3.17 × 10^-3 |
| 2 | Number of cases of ILI per 100,000 population | 5.01 × 10^-4 | 2.58 × 10^-4 | 7.44 × 10^-4 |
| 2 | Proportion of children | 3.95 | 1.99 × 10^-1 | 7.69 |
| 2 | Proportion of males | 5.05 × 10^-1 | -1.49 × 10^1 | 1.60 × 10^1 |
| 2 | Minimum temperature (°C) | 6.02 × 10^-2 | 3.45 × 10^-2 | 8.59 × 10^-2 |
| 2 | Minimum temperature lag-1 (°C) | 6.97 × 10^-2 | 3.92 × 10^-2 | 1.00 × 10^-1 |
| 2 | Minimum temperature lag-2 (°C) | -3.18 × 10^-2 | -5.68 × 10^-2 | -6.90 × 10^-3 |
| 2 | Relative humidity (%) | 2.00 × 10^-2 | 7.60 × 10^-3 | 3.25 × 10^-2 |
| 2 | Relative humidity lag-1 (%) | -7.89 × 10^-3 | -1.63 × 10^-2 | 5.79 × 10^-4 |
| 2 | Relative humidity lag-2 (%) | 6.67 × 10^-3 | 1.89 × 10^-4 | 1.31 × 10^-2 |
| 2 | Rainfall (mm) | 1.06 × 10^-4 | -2.76 × 10^-4 | 4.87 × 10^-4 |
| 2 | Rainfall lag-1 (mm) | 1.99 × 10^-4 | -2.05 × 10^-4 | 6.02 × 10^-4 |
| 2 | Rainfall lag-2 (mm) | -1.34 × 10^-4 | -5.28 × 10^-4 | 2.60 × 10^-4 |
| 2 | Rainfall lag-3 (mm) | -1.36 × 10^-4 | -5.14 × 10^-4 | 2.42 × 10^-4 |
| 2 | Sunshine (hours) | 1.65 × 10^-3 | 6.52 × 10^-4 | 2.65 × 10^-3 |
| 2 | Sunshine lag-1 (hours) | 5.42 × 10^-5 | -9.40 × 10^-4 | 1.05 × 10^-3 |
| 2 | Sunshine lag-2 (hours) | -2.40 × 10^-3 | -3.36 × 10^-3 | -1.44 × 10^-3 |
| 2 | Elevation (m) | 1.13 × 10^-3 | 1.60 × 10^-5 | 2.25 × 10^-3 |
| 2 | NDVI | -3.10 × 10^-2 | -2.68 × 10^-1 | 2.06 × 10^-1 |
| 2 | NDVI lag-1 | 3.93 × 10^-1 | 1.58 × 10^-1 | 6.28 × 10^-1 |
| 2 | NDVI lag-2 | 1.59 × 10^-1 | -7.83 × 10^-2 | 3.97 × 10^-1 |
| 2 | Number of hospitals per 100 km^2^ | -1.09 × 10^-1 | -4.74 × 10^-1 | 2.55 × 10^-1 |
| 2 | Poverty (%) | -2.46 × 10^-2 | -3.52 × 10^-2 | -1.41 × 10^-2 |
| 2 | JEV vaccination coverage (%) | 2.28 × 10^-3 | 1.17 × 10^-3 | 3.39 × 10^-3 |
| 2 | Standardised number of pigs per 100,000 population | 1.82 × 10^-1 | 8.57 × 10^-2 | 2.78 × 10^-1 |
| 3 | (Intercept) | -1.81 × 10^1 | -2.59 × 10^1 | -1.04 × 10^1 |
| 3 | sin(2 * pi * month2/12) | 5.08 × 10^-2 | -2.38 × 10^-1 | 3.37 × 10^-1 |
| 3 | cos(2 * pi * month2/12) | -1.40 × 10^-1 | -4.02 × 10^-1 | 1.26 × 10^-1 |
| 3 | Number of cases of meningitis per 100,000 population | 7.85 × 10^-1 | 6.12 × 10^-1 | 9.59 × 10^-1 |
| 3 | Number of cases of dengue fever per 100,000 population | 1.44 × 10^-3 | -1.67 × 10^-4 | 3.05 × 10^-3 |
| 3 | Number of cases of ILI per 100,000 population | 5.25 × 10^-4 | 2.84 × 10^-4 | 7.67 × 10^-4 |
| 3 | Proportion of children | 4.25 | 4.97 × 10^-1 | 7.99 |
| 3 | Proportion of males | 1.31 | -1.41 × 10^1 | 1.67 × 10^1 |
| 3 | Absolute humidity (g/m3) | 3.97 × 10^-2 | 1.95 × 10^-2 | 6.00 × 10^-2 |
| 3 | Absolute humidity lag-1 (g/m3) | 5.02 × 10^-2 | 2.58 × 10^-2 | 7.47 × 10^-2 |
| 3 | Absolute humidity lag-2 (g/m3) | -2.19 × 10^-2 | -4.28 × 10^-2 | -9.14 × 10^-4 |
| 3 | Relative humidity (%) | 1.08 × 10^-2 | -2.01 × 10^-3 | 2.37 × 10^-2 |
| 3 | Relative humidity lag-1 (%) | -7.98 × 10^-3 | -1.67 × 10^-2 | 7.31 × 10^-4 |
| 3 | Relative humidity lag-2 (%) | 5.96 × 10^-3 | -6.67 × 10^-4 | 1.26 × 10^-2 |
| 3 | Rainfall (mm) | 1.05 × 10^-4 | -2.79 × 10^-4 | 4.90 × 10^-4 |
| 3 | Rainfall lag-1 (mm) | 1.13 × 10^-4 | -2.91 × 10^-4 | 5.16 × 10^-4 |
| 3 | Rainfall lag-2 (mm) | -2.55 × 10^-5 | -4.24 × 10^-4 | 3.73 × 10^-4 |
| 3 | Rainfall lag-3 (mm) | -3.08 × 10^-5 | -4.07 × 10^-4 | 3.45 × 10^-4 |
| 3 | Sunshine (hours) | 1.60 × 10^-3 | 5.65 × 10^-4 | 2.63 × 10^-3 |
| 3 | Sunshine lag-1 (hours) | 1.55 × 10^-4 | -8.42 × 10^-4 | 1.15 × 10^-3 |
| 3 | Sunshine lag-2 (hours) | -2.36 × 10^-3 | -3.31 × 10^-3 | -1.41 × 10^-3 |
| 3 | Elevation (m) | 9.90 × 10^-4 | -1.14 × 10^-4 | 2.10 × 10^-3 |
| 3 | NDVI | -4.08 × 10^-2 | -2.79 × 10^-1 | 1.97 × 10^-1 |
| 3 | NDVI lag-1 | 3.94 × 10^-1 | 1.58 × 10^-1 | 6.29 × 10^-1 |
| 3 | NDVI lag-2 | 1.63 × 10^-1 | -7.45 × 10^-2 | 4.01 × 10^-1 |
| 3 | Standardised number of pigs per 100,000 population | 1.72 × 10^-1 | 7.54 × 10^-2 | 2.68 × 10^-1 |
| 3 | Number of hospitals per 100 km2 | -8.85 × 10^-2 | -4.51 × 10^-1 | 2.73 × 10^-1 |
| 3 | Poverty (%) | -2.37 × 10^-2 | -3.42 × 10^-2 | -1.31 × 10^-2 |
| 3 | JEV vaccination coverage (%) | 2.33 × 10^-3 | 1.22 × 10^-3 | 3.44 × 10^-3 |
| 4 | (Intercept) | -2.04 × 10^1 | -2.82 × 10^1 | -1.27 × 10^1 |
| 4 | sin(2 * pi * month2/12) | 2.36 × 10^-2 | -2.76 × 10^-1 | 3.22 × 10^-1 |
| 4 | cos(2 * pi * month2/12) | -1.33 × 10^-1 | -4.07 × 10^-1 | 1.46 × 10^-1 |
| 4 | Number of cases of meningitis per 100,000 population | 7.86 × 10^-1 | 6.13 × 10^-1 | 9.59 × 10^-1 |
| 4 | Number of cases of dengue fever per 100,000 population | 1.50 × 10^-3 | -1.13 × 10^-4 | 3.12 × 10^-3 |
| 4 | Number of cases of ILI per 100,000 population | 4.90 × 10^-4 | 2.49 × 10^-4 | 7.31 × 10^-4 |
| 4 | Number of cases of S. suis | 2.20 | 1.01 | 3.40 |
| 4 | Number of cases of HFMD per 100,000 population | -6.58 × 10^-3 | -1.13 × 10^-2 | -1.92 × 10^-3 |
| 4 | Proportion of children | 4.15 | 4.04 × 10^-1 | 7.88 |
| 4 | Proportion of males | 1.54 | -1.38 × 10^1 | 1.69 × 10^1 |
| 4 | Maximum temperature (°C) | 6.67 × 10^-2 | 4.04 × 10^-2 | 9.30 × 10^-2 |
| 4 | Maximum temperature lag-1 (°C) | 6.08 × 10^-2 | 3.16 × 10^-2 | 9.00 × 10^-2 |
| 4 | Maximum temperature lag-2 (°C) | -3.42 × 10^-2 | -5.77 × 10^-2 | -1.06 × 10^-2 |
| 4 | Relative humidity (%) | 2.80 × 10^-2 | 1.55 × 10^-2 | 4.06 × 10^-2 |
| 4 | Relative humidity lag-1 (%) | -7.94 × 10^-3 | -1.66 × 10^-2 | 7.32 × 10^-4 |
| 4 | Relative humidity lag-2 (%) | 7.12 × 10^-3 | 3.38 × 10^-4 | 1.39 × 10^-2 |
| 4 | Rainfall (mm) | 1.46 × 10^-4 | -2.37 × 10^-4 | 5.29 × 10^-4 |
| 4 | Rainfall lag-1 (mm) | 3.41 × 10^-4 | -6.29 × 10^-5 | 7.44 × 10^-4 |
| 4 | Rainfall lag-2 (mm) | -7.97 × 10^-5 | -4.71 × 10^-4 | 3.11 × 10^-4 |
| 4 | Rainfall lag-3 (mm) | -4.33 × 10^-5 | -4.15 × 10^-4 | 3.28 × 10^-4 |
| 4 | Sunshine (hours) | 7.96 × 10^-4 | -3.63 × 10^-4 | 1.95 × 10^-3 |
| 4 | Sunshine lag-1 (hours) | -7.34 × 10^-4 | -1.93 × 10^-3 | 4.59 × 10^-4 |
| 4 | Sunshine lag-2 (hours) | -1.70 × 10^-3 | -2.89 × 10^-3 | -5.18 × 10^-4 |
| 4 | Elevation (m) | 8.39 × 10^-4 | -2.43 × 10^-4 | 1.93 × 10^-3 |
| 4 | NDVI | -8.20 × 10^-2 | -3.20 × 10^-1 | 1.56 × 10^-1 |
| 4 | NDVI lag-1 | 3.42 × 10^-1 | 1.06 × 10^-1 | 5.78 × 10^-1 |
| 4 | NDVI lag-2 | 1.45 × 10^-1 | -9.34 × 10^-2 | 3.83 × 10^-1 |
| 4 | Standardised number of pigs per 100,000 population | 1.61 × 10^-1 | 6.39 × 10^-2 | 2.58 × 10^-1 |
| 4 | Number of hospitals per 100 km^2^ | -1.36 × 10^-1 | -4.98 × 10^-1 | 2.26 × 10^-1 |
| 4 | Poverty (%) | -2.46 × 10^-2 | -3.52 × 10^-2 | -1.41 × 10^-2 |
| 4 | JEV vaccination coverage (%)3 | 2.50 × 10^-3 | 1.39 × 10^-3 | 3.61 × 10^-3 |
| 5 | (Intercept) | -1.95 × 10^1 | -2.72 × 10^1 | -1.17 × 10^1 |
| 5 | sin(2 * pi * month2/12) | 1.38 × 10^-2 | -2.63 × 10^-1 | 2.89 × 10^-1 |
| 5 | cos(2 * pi * month2/12) | -1.23 × 10^-1 | -3.76 × 10^-1 | 1.33 × 10^-1 |
| 5 | Number of cases of meningitis per 100,000 population | 7.87 × 10^-1 | 6.14 × 10^-1 | 9.60 × 10^-1 |
| 5 | Number of cases of dengue fever per 100,000 population | 1.54 × 10^-3 | -8.49 × 10^-5 | 3.16 × 10^-3 |
| 5 | Number of cases of ILI per 100,000 population | 4.99 × 10^-4 | 2.57 × 10^-4 | 7.42 × 10^-4 |
| 5 | Number of cases of *S. suis* | 2.16 | 9.81 × 10^-1 | 3.33 |
| 5 | Number of cases of HFMD per 100,000 population | -6.49 × 10^-3 | -1.11 × 10^-2 | -1.83 × 10^-3 |
| 5 | Proportion of children | 4.03 | 2.85 × 10^-1 | 7.75 |
| 5 | Proportion of males | 1.67 | -1.37 × 10^1 | 1.71 × 10^1 |
| 5 | Minimum temperature (°C) | 6.15 × 10^-2 | 3.59 × 10^-2 | 8.72 × 10^-2 |
| 5 | Minimum temperature lag-1 (°C) | 6.87 × 10^-2 | 3.83 × 10^-2 | 9.91 × 10^-2 |
| 5 | Minimum temperature lag-2 (°C) | -3.57 × 10^-2 | -6.07 × 10^-2 | -1.07 × 10^-2 |
| 5 | Relative humidity (%) | 2.06 × 10^-2 | 8.23 × 10^-3 | 3.30 × 10^-2 |
| 5 | Relative humidity lag-1 (%) | -7.74 × 10^-3 | -1.62 × 10^-2 | 7.09 × 10^-4 |
| 5 | Relative humidity lag-2 (%) | 7.30 × 10^-3 | 8.26 × 10^-4 | 1.38 × 10^-2 |
| 5 | Rainfall (mm) | 1.09 × 10^-4 | -2.72 × 10^-4 | 4.90 × 10^-4 |
| 5 | Rainfall lag-1 (mm) | 2.00 × 10^-4 | -2.02 × 10^-4 | 6.03 × 10^-4 |
| 5 | Rainfall lag-2 (mm) | -1.53 × 10^-4 | -5.47 × 10^-4 | 2.40 × 10^-4 |
| 5 | Rainfall lag-3 (mm) | -1.20 × 10^-4 | -4.97 × 10^-4 | 2.58 × 10^-4 |
| 5 | Sunshine (hours) | 1.68 × 10^-3 | 6.81 × 10^-4 | 2.67 × 10^-3 |
| 5 | Sunshine lag-1 (hours) | 1.06 × 10^-4 | -8.87 × 10^-4 | 1.10 × 10^-3 |
| 5 | Sunshine lag-2 (hours) | -2.28 × 10^-3 | -3.24 × 10^-3 | -1.32 × 10^-3 |
| 5 | Elevation (m) | 1.09 × 10^-3 | -2.10 × 10^-5 | 2.19 × 10^-3 |
| 5 | NDVI | -5.27 × 10^-2 | -2.90 × 10^-1 | 1.84 × 10^-1 |
| 5 | NDVI lag-1 | 3.88 × 10^-1 | 1.54 × 10^-1 | 6.23 × 10^-1 |
| 5 | NDVI lag-2 | 1.55 × 10^-1 | -8.17 × 10^-2 | 3.93 × 10^-1 |
| 5 | Standardised number of pigs per 100,000 population | 1.76 × 10^-1 | 7.96 × 10^-2 | 2.72 × 10^-1 |
| 5 | Number of hospitals per 100 km^2^ | -1.10 × 10^-1 | -4.73 × 10^-1 | 2.53 × 10^-1 |
| 5 | Poverty (%) | -2.44 × 10^-2 | -3.49 × 10^-2 | -1.39 × 10^-2 |
| 5 | JEV vaccination coverage (%) | 2.42 × 10^-3 | 1.31 × 10^-3 | 3.53 × 10^-3 |
| 6 | (Intercept) | -1.87 × 10^1 | -2.65 × 10^1 | -1.10 × 10^1 |
| 6 | sin(2 * pi * month2/12) | 2.91 × 10^-2 | -2.68 × 10^-1 | 3.23 × 10^-1 |
| 6 | cos(2 * pi * month2/12) | -1.34 × 10^-1 | -4.02 × 10^-1 | 1.40 × 10^-1 |
| 6 | Number of cases of meningitis per 100,000 population | 7.85 × 10^-1 | 6.12 × 10^-1 | 9.59 × 10^-1 |
| 6 | Number of cases of dengue fever per 100,000 population | 1.44 × 10^-3 | -1.68 × 10^-4 | 3.05 × 10^-3 |
| 6 | Number of cases of ILI per 100,000 population | 5.25 × 10^-4 | 2.84 × 10^-4 | 7.67 × 10^-4 |
| 6 | Number of cases of *S. suis* | 2.13 | 9.48 × 10^-1 | 3.31 |
| 6 | Number of cases of HFMD per 100,000 population | -6.48 × 10^-3 | -1.12 × 10^-2 | -1.80 × 10^-3 |
| 6 | Proportion of children | 4.29 | 5.39 × 10^-1 | 8.03 |
| 6 | Proportion of males | 2.39 | -1.30 × 10^1 | 1.78 × 10^1 |
| 6 | Absolute humidity (g/m3) | 4.12 × 10^-2 | 2.10 × 10^-2 | 6.15 × 10^-2 |
| 6 | Absolute humidity lag-1 (g/m3) | 4.89 × 10^-2 | 2.44 × 10^-2 | 7.33 × 10^-2 |
| 6 | Absolute humidity lag-2 (g/m3) | -2.61 × 10^-2 | -4.72 × 10^-2 | -5.08 × 10^-3 |
| 6 | Relative humidity (%) | 1.13 × 10^-2 | -1.56 × 10^-3 | 2.41 × 10^-2 |
| 6 | Relative humidity lag-1 (%) | -7.61 × 10^-3 | -1.63 × 10^-2 | 1.10 × 10^-3 |
| 6 | Relative humidity lag-2 (%) | 6.76 × 10^-3 | 1.28 × 10^-4 | 1.34 × 10^-2 |
| 6 | Rainfall (mm) | 1.06 × 10^-4 | -2.78 × 10^-4 | 4.90 × 10^-4 |
| 6 | Rainfall lag-1 (mm) | 1.18 × 10^-4 | -2.86 × 10^-4 | 5.21 × 10^-4 |
| 6 | Rainfall lag-2 (mm) | -2.88 × 10^-5 | -4.27 × 10^-4 | 3.70 × 10^-4 |
| 6 | Rainfall lag-3 (mm) | -7.95 × 10^-6 | -3.84 × 10^-4 | 3.68 × 10^-4 |
| 6 | Sunshine (hours) | 1.65 × 10^-3 | 6.13 × 10^-4 | 2.68 × 10^-3 |
| 6 | Sunshine lag-1 (hours) | 2.28 × 10^-4 | -7.69 × 10^-4 | 1.22 × 10^-3 |
| 6 | Sunshine lag-2 (hours) | -2.21 × 10^-3 | -3.16 × 10^-3 | -1.25 × 10^-3 |
| 6 | Elevation (m) | 9.35 × 10^-4 | -1.62 × 10^-4 | 2.03 × 10^-3 |
| 6 | NDVI | -6.08 × 10^-2 | -2.98 × 10^-1 | 1.77 × 10^-1 |
| 6 | NDVI lag-1 | 3.91 × 10^-1 | 1.55 × 10^-1 | 6.26 × 10^-1 |
| 6 | NDVI lag-2 | 1.62 × 10^-1 | -7.54 × 10^-2 | 4.00 × 10^-1 |
| 6 | Standardised number of pigs per 100,000 population | 1.65 × 10^-1 | 6.84 × 10^-2 | 2.62 × 10^-1 |
| 6 | Number of hospitals per 100 km^2^ | -9.39 × 10^-2 | -4.55 × 10^-1 | 2.67 × 10^-1 |
| 6 | Poverty (%) | -2.35 × 10^-2 | -3.41 × 10^-2 | -1.30 × 10^-2 |
| 6 | JEV vaccination coverage (%) | 2.45 × 10^-3 | 1.34 × 10^-3 | 3.56 × 10^-3 |
| 7 | (Intercept) | -1.88 × 10^1 | -2.67 × 10^1 | -1.10 × 10^1 |
| 7 | sin(2 * pi * month2/12) | 1.94 × 10^-2 | -2.99 × 10^-1 | 3.36 × 10^-1 |
| 7 | cos(2 * pi * month2/12 | -1.46 × 10^-1 | -4.38 × 10^-1 | 1.50 × 10^-1 |
| 7 | Proportion of children | 3.32 | -4.33 × 10^-1 | 7.08 |
| 7 | Proportion of males | -1.45 | -1.70 × 10^1 | 1.41 × 10^1 |
| 7 | Maximum temperature (°C) | 7.00 × 10^-2 | 4.35 × 10^-2 | 9.64 × 10^-2 |
| 7 | Maximum temperature lag-1 (°C) | 6.25 × 10^-2 | 3.31 × 10^-2 | 9.19 × 10^-2 |
| 7 | Maximum temperature lag-2 (°C) | -3.10 × 10^-2 | -5.46 × 10^-2 | -7.46 × 10^-3 |
| 7 | Relative humidity (%) | 2.89 × 10^-2 | 1.63 × 10^-2 | 4.16 × 10^-2 |
| 7 | Relative humidity lag-1 (%) | -7.08 × 10^-3 | -1.58 × 10^-2 | 1.63 × 10^-3 |
| 7 | Relative humidity lag-2 (%) | 5.95 × 10^-3 | -8.45 × 10^-4 | 1.27 × 10^-2 |
| 7 | Rainfall (mm) | 7.13 × 10^-5 | -3.12 × 10^-4 | 4.54 × 10^-4 |
| 7 | Rainfall lag-1 (mm) | 4.25 × 10^-4 | 2.02 × 10^-5 | 8.31 × 10^-4 |
| 7 | Rainfall lag-2 (mm) | -6.77 × 10^-5 | -4.61 × 10^-4 | 3.25 × 10^-4 |
| 7 | Rainfall lag-3 (mm) | -3.42 × 10^-5 | -4.07 × 10^-4 | 3.39 × 10^-4 |
| 7 | Sunshine (hours) | 6.41 × 10^-4 | -5.24 × 10^-4 | 1.81 × 10^-3 |
| 7 | Sunshine lag-1 (hours) | -7.89 × 10^-4 | -1.99 × 10^-3 | 4.11 × 10^-4 |
| 7 | Sunshine lag-2 (hours) | -1.87 × 10^-3 | -3.06 × 10^-3 | -6.80 × 10^-4 |
| 7 | Elevation (m) | 1.19 × 10^-3 | 1.01 × 10^-4 | 2.30 × 10^-3 |
| 7 | NDVI | -6.54 × 10^-2 | -3.04 × 10^-1 | 1.74 × 10^-1 |
| 7 | NDVI lag-1 | 3.61 × 10^-1 | 1.24 × 10^-1 | 5.98 × 10^-1 |
| 7 | NDVI lag-2 | 1.52 × 10^-1 | -8.77 × 10^-2 | 3.92 × 10^-1 |
| 7 | Number of hospitals per 100 km^2^ | -1.34 × 10^-1 | -5.00 × 10^-1 | 2.30 × 10^-1 |
| 7 | Poverty (%) | -2.30 × 10^-2 | -3.38 × 10^-2 | -1.24 × 10^-2 |
| 7 | JEV vaccination coverage (%) | 1.99 × 10^-3 | 8.74 × 10^-4 | 3.10 × 10^-3 |
| 7 | Standardised number of pigs per 100,000 population | 1.68 × 10^-1 | 7.12 × 10^-2 | 2.64 × 10^-1 |
| 8 | (Intercept) | -1.78 × 10^1 | -2.56 × 10^1 | -9.92 |
| 8 | sin(2 * pi * month2/12) | 1.08 × 10^-2 | -3.56 × 10^-1 | 3.65 × 10^-1 |
| 8 | cos(2 * pi * month2/12) | -1.39 × 10^-1 | -4.64 × 10^-1 | 2.02 × 10^-1 |
| 8 | Proportion of children | 3.10 | -6.95 × 10^-1 | 6.90 |
| 8 | Proportion of males | -1.20 | -1.68 × 10^1 | 1.44 × 10^1 |
| 8 | Minimum temperature (°C) | 6.14 × 10^-2 | 3.56 × 10^-2 | 8.74 × 10^-2 |
| 8 | Minimum temperature lag-1 (°C) | 7.39 × 10^-2 | 4.33 × 10^-2 | 1.04 × 10^-1 |
| 8 | Minimum temperature lag-2 (°C) | -3.35 × 10^-2 | -5.86 × 10^-2 | -8.47 × 10^-3 |
| 8 | Relative humidity (%) | 2.12 × 10^-2 | 8.69 × 10^-3 | 3.38 × 10^-2 |
| 8 | Relative humidity lag-1 (%) | -7.55 × 10^-3 | -1.61 × 10^-2 | 1.02 × 10^-3 |
| 8 | Relative humidity lag-2 (%) | 6.54 × 10^-3 | 4.44 × 10^-5 | 1.30 × 10^-2 |
| 8 | Rainfall (mm) | 3.57 × 10^-5 | -3.46 × 10^-4 | 4.18 × 10^-4 |
| 8 | Rainfall lag-1 (mm) | 2.92 × 10^-4 | -1.15 × 10^-4 | 6.97 × 10^-4 |
| 8 | Rainfall lag-2 (mm) | -1.40 × 10^-4 | -5.35 × 10^-4 | 2.55 × 10^-4 |
| 8 | Rainfall lag-3 (mm) | -1.25 × 10^-4 | -5.04 × 10^-4 | 2.54 × 10^-4 |
| 8 | Sunshine (hours) | 1.59 × 10^-3 | 5.86 × 10^-4 | 2.59 × 10^-3 |
| 8 | Sunshine lag-1 (hours) | 6.44 × 10^-5 | -9.33 × 10^-4 | 1.06 × 10^-3 |
| 8 | Sunshine lag-2 (hours) | -2.39 × 10^-3 | -3.35 × 10^-3 | -1.42 × 10^-3 |
| 8 | Elevation (m) | 1.45 × 10^-3 | 3.33 × 10^-4 | 2.57 × 10^-3 |
| 8 | NDVI | -3.53 × 10^-2 | -2.73 × 10^-1 | 2.03 × 10^-1 |
| 8 | NDVI lag-1 | 4.06 × 10^-1 | 1.70 × 10^-1 | 6.42 × 10^-1 |
| 8 | NDVI lag-2 | 1.63 × 10^-1 | -7.55 × 10^-2 | 4.02 × 10^-1 |
| 8 | Number of hospitals per 100 km^2^ | -1.30 × 10^-1 | -4.99 × 10^-1 | 2.36 × 10^-1 |
| 8 | Poverty (%) | -2.32 × 10^-2 | -3.40 × 10^-2 | -1.25 × 10^-2 |
| 8 | JEV vaccination coverage (%) | 1.89 × 10^-3 | 7.74 × 10^-4 | 3.00 × 10^-3 |
| 8 | Standardised number of pigs per 100,000 populatio | 1.76 × 10^-1 | 7.83 × 10^-2 | 2.73 × 10^-1 |
| 9 | (Intercept) | -1.71 × 10^1 | -2.49 × 10^1 | -9.24 |
| 9 | sin(2 * pi * month2/12) | 4.50 × 10^-2 | -2.44 × 10^-1 | 3.31 × 10^-1 |
| 9 | cos(2 * pi * month2/12) | -1.47 × 10^-1 | -4.09 × 10^-1 | 1.19 × 10^-1 |
| 9 | Proportion of children | 3.44 | -4.09 × 10^-1 | 7.29 |
| 9 | Proportion of males | -3.89 × 10^-1 | -1.60 × 10^1 | 1.52 × 10^1 |
| 9 | Absolute humidity (g/m3) | 4.06 × 10^-2 | 2.03 × 10^-2 | 6.10 × 10^-2 |
| 9 | Absolute humidity lag-1 (g/m3) | 5.42 × 10^-2 | 2.97 × 10^-2 | 7.87 × 10^-2 |
| 9 | Absolute humidity lag-2 (g/m3) | -2.18 × 10^-2 | -4.29 × 10^-2 | -7.12 × 10^-4 |
| 9 | Relative humidity (%) | 1.20 × 10^-2 | -9.75 × 10^-4 | 2.49 × 10^-2 |
| 9 | Relative humidity lag-1 (%) | -7.87 × 10^-3 | -1.66 × 10^-2 | 8.62 × 10^-4 |
| 9 | Relative humidity lag-2 (%) | 5.56 × 10^-3 | -1.07 × 10^-3 | 1.22 × 10^-2 |
| 9 | Rainfall (mm) | 3.79 × 10^-5 | -3.46 × 10^-4 | 4.22 × 10^-4 |
| 9 | Rainfall lag-1 (mm) | 1.94 × 10^-4 | -2.10 × 10^-4 | 5.98 × 10^-4 |
| 9 | Rainfall lag-2 (mm) | -3.07 × 10^-5 | -4.31 × 10^-4 | 3.70 × 10^-4 |
| 9 | Rainfall lag-3 (mm) | -1.63 × 10^-5 | -3.94 × 10^-4 | 3.61 × 10^-4 |
| 9 | Sunshine (hours) | 1.53 × 10^-3 | 4.96 × 10^-4 | 2.57 × 10^-3 |
| 9 | Sunshine lag-1 (hours) | 1.57 × 10^-4 | -8.44 × 10^-4 | 1.16 × 10^-3 |
| 9 | Sunshine lag-2 (hours) | -2.35 × 10^-3 | -3.31 × 10^-3 | -1.40 × 10^-3 |
| 9 | Elevation (m) | 1.30 × 10^-3 | 1.90 × 10^-4 | 2.41 × 10^-3 |
| 9 | NDVI | -5.09 × 10^-2 | -2.90 × 10^-1 | 1.88 × 10^-1 |
| 9 | NDVI lag-1 | 4.00 × 10^-1 | 1.63 × 10^-1 | 6.36 × 10^-1 |
| 9 | NDVI lag-2 | 1.62 × 10^-1 | -7.73 × 10^-2 | 4.01 × 10^-1 |
| 9 | Standardised number of pigs per 100,000 population | 1.53 × 10^-1 | 5.52 × 10^-2 | 2.52 × 10^-1 |
| 9 | Number of hospitals per 100 km^2^ | -1.45 × 10^-1 | -5.12 × 10^-1 | 2.21 × 10^-1 |
| 9 | Poverty (%) | -2.29 × 10^-2 | -3.35 × 10^-2 | -1.22 × 10^-2 |
| 9 | JEV vaccination coverage (%) | 1.91 × 10^-3 | 7.91 × 10^-4 | 3.02 × 10^-3 |
